# Unstable Relationship Between *Braarudosphaera bigelowii* (= *Chrysochromulina parkeae*) and Its Nitrogen-Fixing Endosymbiont

**DOI:** 10.3389/fpls.2021.749895

**Published:** 2021-12-03

**Authors:** Shigekatsu Suzuki, Masanobu Kawachi, Chinatsu Tsukakoshi, Atsushi Nakamura, Kyoko Hagino, Isao Inouye, Ken-ichiro Ishida

**Affiliations:** ^1^Biodiversity Division, National Institute for Environmental Studies, Ibaraki, Japan; ^2^Graduate School of Life and Environmental Sciences, University of Tsukuba, Ibaraki, Japan; ^3^Center for Advanced Marine Core Research, Kochi University, Kochi, Japan; ^4^Faculty of Life and Environmental Sciences, University of Tsukuba, Ibaraki, Japan

**Keywords:** nitrogen fixation, endosymbiosis, haptophyte, UCYN-A, reductive evolution

## Abstract

Marine phytoplankton are major primary producers, and their growth is primarily limited by nitrogen in the oligotrophic ocean environment. The haptophyte *Braarudosphaera bigelowii* possesses a cyanobacterial endosymbiont (UCYN-A), which plays a major role in nitrogen fixation in the ocean. However, host-symbiont interactions are poorly understood because *B. bigelowii* was unculturable. In this study, we sequenced the complete genome of the *B. bigelowii* endosymbiont and showed that it was highly reductive and closely related to UCYN-A2 (an ecotype of UCYN-A). We succeeded in establishing *B. bigelowii* strains and performed microscopic observations. The detailed observations showed that the cyanobacterial endosymbiont was surrounded by a single host derived membrane and divided synchronously with the host cell division. The transcriptome of *B. bigelowii* revealed that *B. bigelowii* lacked the expression of many essential genes associated with the uptake of most nitrogen compounds, except ammonia. During cultivation, some of the strains completely lost the endosymbiont. Moreover, we did not find any evidence of endosymbiotic gene transfer from the endosymbiont to the host. These findings illustrate an unstable morphological, metabolic, and genetic relationship between *B. bigelowii* and its endosymbiont.

## Introduction

Marine phytoplankton consume various nitrogen compounds, such as nitrate, nitrite, ammonia, and cyanate, and often produce large-scale blooms triggered by the abundance of specific nitrogen compounds (Suzuki et al., 2019). In oligotrophic ocean environments, phytoplankton growth is primarily limited by the availability of nitrogen compounds. Atmospheric nitrogen (N_2_) is fixed by some cyanobacteria (diazotrophy; Galloway et al., 2004) that possess the nitrogenase enzyme, encoded by the *nif* gene cluster (Tsygankov, 2007). Long-term monitoring of ocean diazotroph community structure and nitrogen fixation rates revealed that unicellular cyanobacterial species contribute more to nitrogen fixation in oceans than filamentous species, such as *Trichodesmium* spp. (Church et al., 2009). In the unicellular species, UCYN-A (unicellular N_2_-fixing cyanobacteria group A), which is also described as *Candidatus* Atelocyanobacterium thalassa, was initially identified based on environmental sequences of *nifH* (Zehr et al., 2001). To date, the cultivation of this species has been unsuccessful. UCYN-A consists of at least six sub-lineages, UCYN-A1 to UCYN-A6, which have different ecological niches (Thompson et al., 2014; Farnelid et al., 2016; Turk-Kubo et al., 2017). Among the sub-lineages, UCYN-A2 has been commonly reported in coastal waters (Turk-Kubo et al., 2017), although it has also been reported in the open ocean.

The exchange of metabolites between UCYN-A (UCYN-A1 and UCYN-A2) and a prymnesiophyte, *Braarudosphaera bigelowii* (haptophyte), was revealed (Thompson et al., 2012; Martínez-Pérez et al., 2016; Mills et al., 2020); UCYN-A1 and UCYN-A2 provide fixed nitrogen to *B. bigelowii* and, in exchange, receive fixed carbon from *B. bigelowii*. Recently, Hagino et al. (2013) reported an endosymbiotic UCYN-A2 in the calcareous coccolithophore *B. bigelowii*. The endosymbiont was separated from its host cell by a single membrane in *B. bigelowii* (Hagino et al., 2013, 2016). However, host-symbiont interactions remain unclear because *B. bigelowii* is unculturable.

The UCYN-A genome lacks many essential genes required for survival as a free-living organism (Zehr et al., 2008; Tripp et al., 2010). The complete genome of the ecotype UCYN-A1 was sequenced by whole-genome amplification (WGA) of cells purified by flow cytometry (Zehr et al., 2008; Tripp et al., 2010). The genome was highly reduced (1.44 Mbp) and contained genes for nitrogen fixation, but lacked some vital metabolic pathways, such as photosystem II, Calvin cycle, and the tricarboxylic acid (TCA) cycle. Genes for photosystem I were retained, which could provide electrons for nitrogenase. The loss of photosystem II may be advantageous because UCYN-A expresses nitrogenase (Church et al., 2005), which is inactivated by oxygen (Zehr et al., 2017) during daylight hours. Subsequently, the draft genome of UCYN-A2 was sequenced using a similar WGA method (i.e., using cells isolated by flow cytometry; Bombar et al., 2014). The genomes of UCYN-A1 and UCYN-A2 share most of the protein-coding genes (96.6%) with high synteny but high amino acid diversity. The host of UCYN-A2 is significantly larger in cell size than that of UCYN-A1 (Thompson et al., 2014). Only UCYN-A2 possesses genes for cell shape and cell wall biogenesis, suggesting different associations between the two UCYN-A ecotypes and their hosts (Bombar et al., 2014).

*Braarudosphaera bigelowii* is a species complex consisting of multiple pseudo-cryptic species based on genetic and morphological divergence (Hagino et al., 2009). In this species complex, 18S rDNA genotype III is considered *B. bigelowii sensu stricto* (s.s.). *B. bigelowii* s.s. differed from *Chrysochromulina parkeae* in morphology but was almost identical to *C. parkeae* in the 18S rDNA sequence (99.89% similarity). Therefore, *C. parkeae* is considered an alternate life cycle stage in *B. bigelowii* s.s. (Hagino et al., 2013). In the present study, we sequenced an endosymbiont genome in the *C. parkeae* stage of *B. bigelowii*. We established *B. bigelowii* strains and observed morphological interactions between *B. bigelowii* and its endosymbiont. Transcriptome analyses were also performed to elucidate nitrogen metabolism in *B. bigelowii*.

## Materials and Methods

### Sampling

Sea surface water was collected at Asamushi, Aomori, Japan (40°53′37.2″N 140°51′32.5″E) in 1990, at Tomari Port, Tottori, Japan (35°31′01.7″N 133°56′14.8″E) between 2013 and 2014, and at Ikenoura Port, Kochi, Japan (33°24′34.9″N 133°24′45.0″E) between 2015 and 2017. Detailed information on the samples is provided in **Supplementary Material** (Supplementary Table 1).

### Genome Sequencing of the *B. bigelowii* Endosymbiont

Sea surface water collected from Tomari Port in 2014 was used to sequence the genome of the endosymbiont of the *C. parkeae* stage of *B. bigelowii*. *B. bigelowii* was temporarily maintained in ESM medium (Kasai et al., 2009) with 0.25mg/l GeO_2_. A single *B. bigelowii* cell was isolated and washed once with distilled water by micropipetting under an inverted microscope. The cell was frozen at −30°C to disrupt the cell membrane. DNA in the lysate was amplified using an Illustra GenomiPhi DNA Amplification kit (GE Healthcare, Piscataway, NJ, United States), and single-stranded DNA was digested with S1 nuclease (Takara, Shiga, Japan). The amplified DNA was purified using a phenol:chloroform:isoamyl alcohol (25:24:1) and chloroform:isoamyl alcohol (24:1) mixture. Subsequently, DNA was concentrated by ethanol precipitation from the aqueous phase.

DNA was sequenced using the Illumina MiSeq platform (Illumina, San Diego, CA, United States) with a 300bp×2 library (Fasmac, Kanagawa, Japan). The read sequences were deposited in DDBJ/GenBank/ENA under the accession number DRA011127. We obtained 7,469,122 reads (2.1 Gbp) and removed adapter sequences using TagCleaner version 0.16 (Schmieder et al., 2010). Reads of <50bp and the 5′ and 3′ ends with quality less than Q20 were trimmed using PRINSEQ version 0.20.4 (Schmieder and Edwards, 2011). The trimmed reads were assembled into 8,895 scaffolds using SPAdes 3.1.1, with a k-mer value of 77 (Bankevich et al., 2012). The 188 scaffolds that originated from the endosymbiont were selected by homology search using blastn against the genome of *Candidatus* Atelocyanobacterium thalassa isolate ALOHA. The scaffolds were re-assembled into four major contigs using CodonCode aligner version 3.7.1.1 (CodonCode, Centerville, MA, United States). All remaining gaps were closed by PCR using TaKaRa Ex Taq (Takara). The PCR products were ligated into the pGEM-T easy vector (Promega, Madison, WI, United States). The plasmids were sequenced with an ABI 3130 sequencer (Applied Biosystems, Foster City, CA, United States) using the BigDye version 3.1 kit (Applied Biosystems). The Illumina reads were mapped to the genome sequence using Minimap2 version 2.17-r941 (Li, 2018) using the option (−x sr), and the genome sequence was polished with Pilon 1.22 (Walker et al., 2014). The polishing step was repeated three times. Gene models were predicted using Prokka 1.14.0 (Seemann, 2014) with manual curation, and functional annotation was performed using the EggNOG-mapper web server (Huerta-Cepas et al., 2017). Syntenic analysis among the *B. bigelowii* endosymbiont, UCYN-A1, and UCYN-A2 was performed using progressiveMauve 2.4.0 (Darling et al., 2010) with default options. Before the alignment, the contigs of UCYN-A2 were reordered with the “Move Contigs” option, using the endosymbiont genome as a reference. The genome sequence was deposited in DDBJ/GenBank/ENA under accession number AP024987.

### Establishment of *B. bigelowii* Strains

For strains KC1-P2 and KC15-24, seawater samples collected from Ikenoura Port were used to study the *C. parkeae* stage of *B. bigelowii* in culture. Each sample was concentrated using an isopore membrane (pore size, 5.0μm; Merck, Darmstadt, Germany). The concentrated cells were precultured in light–dark cycles consisting of 12h of light and 12h of darkness at 18°C, in 50% ESM medium with 0.75mg/l GeO_2_ and without nitrogen compounds. Single *B. bigelowii* cells were isolated by micropipetting, and each isolate was grown in a culture well under the same conditions. Two isolates from seawater samples collected on May 17, 2015, and May 24, 2017 were labeled as KC1-P2 and KC15-24, respectively (Supplementary Table 1). KC1-P2 possessed an endosymbiont at the beginning of the culture experiments but lost it during the course of the experiments. KC15-24 did not have an endosymbiont at the beginning of the culture experiments. However, it is unknown whether the cell originally lacked the endosymbiont or lost it at the beginning of the preculture. Strains KC1-P2 and KC15-24 were deposited in the National Institute for Environmental Studies (NIES) and are available as strains NIES-3865 and NIES-4442, respectively. Strain MK90-06 was isolated from the seawater sample from Asamushi approximately two decades ago, using a method similar to that used for the seawater sample from Ikenoura Port, and was maintained for roughly a year; however, this strain is now extinct.

### Light Microscopy, Fluorescence Microscopy, and Transmission Electron Microscopy

The KC1-P2 and KC15-24 strains were observed using an Axio Imager.A2 microscope (Carl Zeiss, Berlin, Germany) equipped with an Olympus DP71 or DP74 CCD camera (Olympus, Tokyo, Japan). Endosymbiont-bearing KC1-P2 was observed using fluorescence microscopy. Fixed cells were stained with 4,6-diamidino-2-phenylindole (DAPI) in the dark and mounted with SlowFade DIAMOND (Invitrogen, Carlsbad, CA). Specimens were then observed under a Leica DMRD microscope (Leica, Wetzlar, Germany) equipped with an Olympus DP73 CCD camera (Olympus, Tokyo, Japan). For transmission electron microscopy (TEM) observations of the *C. parkeae* stage of *B. bigelowii* and its endosymbiont, the MK90-06 strain was used. Detailed methods for sample treatment and observation are described in Kawachi et al. (1991) (Supplementary Material).

### Phylogenetic Analysis Using Cyanobacterial Genomes

The dataset was composed of 64 cyanobacterial species, including UCYN-A1, UCYN-A2, UBA4158 (a metagenome assigned as UCYN-A), and the *B. bigelowii* endosymbiont. One hundred and sixty single-copy orthologous proteins were identified using OrthoFinder version 2.1.2 (Emms and Kelly, 2019). The sequences were aligned using MAFFT version 7.453 (Katoh and Toh, 2008) with the auto option. Alignments were trimmed using trimAl version 1.4.rev15 (Capella-Gutierrez et al., 2009) with the option “automated1.” Model testing was performed using ModelTest-NG version 0.1.5 (Darriba et al., 2020). The selected substitution models were shown in Supplementary Table 2. Maximum likelihood (ML) analyses were performed using RAxML-NG version 0.9.0 (Kozlov et al., 2019) with 200 bootstrap replicates.

### Transcriptome Analyses of Endosymbiont-Free *B. bigelowii*

For RNA-seq analyses, endosymbiont-free KC1-P2 cells were used (Supplementary Table 3). The cells were cultivated in the ESM and a nitrogen-free ESM medium. The cells were collected in light and dark phases by gentle centrifugation. RNA was extracted from five independent cultures using TRIzol Reagent (Thermo Fisher Scientific, Waltham, MA, United States) following the manufacturer’s protocol (Supplementary Table 3). mRNA was selected by enriching the polyA sequences and sequenced on a HiSeq 2,500 system (125bp×2; Illumina), which were performed by Eurofins Genomics (Ebersberg, Germany). The paired-end libraries were constructed with ~200bp cDNA inserts and sequenced using TruSeq SBS Kit v3 (250cycle; Illumina). We obtained a total of 26.1 Gbp and 220 million paired-end sequences. Quality of reads was checked and reads with low quality (phred quality <Q15 and length<15bp) were trimmed using fastp 0.20.0 (Chen et al., 2018) with default options. All of the read sets were assembled and clustered using DRAP 1.92 (Cabau et al., 2017) with the Oases assembler (Schulz et al., 2012). The completeness of the assembly was checked with BUSCO version 4.0.6 with the “eukaryote_odb10” database (Simão et al., 2015). The transcript contigs were annotated using the EggNOG-mapper web server. Moreover, we searched homologs of nitrogen metabolism-related genes of *Emiliamia huxleyi* using tblastn with a cutoff: e-value <1E-3. We also confirmed the presence/absence of these genes at the sequenced read level. We merged the paired-end reads of the RNA-seq, and the short reads less than 100bp were removed using fastp (Chen et al., 2018). All read sets were merged, and redundant sequences were clustered using CD-HIT-EST version 4.8.1 (Fu et al., 2012) with the option (−c 1). Using the non-redundant reads as a database, we performed tblastn search with the same method. For gene expression analysis, we mapped each RNA-seq sample to the transcript contigs using minimap2 version 2.20 (Li, 2018) with the option (−x sr). The number of mapped reads was counted using Samtools version 1.5 (Li et al., 2009). RNA-Seq reads were deposited in DDBJ/GenBank/ENA under the accession number DRA011134.

### Prediction of Phagotrophy in *B. bigelowii*

We predict the trophic modes of *B. bigelowii* and a mixotrophic haptophyte *Haptolina brevifila* using the transcriptomes, based on the methods by Burns et al. (2018) and Bock et al. (2021). In this model-based analysis, the probability of potential phagocytosis of organisms is evaluated with the scores of 0 to 1. A probability more than 0.5 is interpreted as presence of specific trophic mode including phagotrophy. We translated the assembled transcripts of *B. bigelowii* into protein sequences using TransDecoder v5.5.0.^1^ Proteins of *H. brevifila* (MMETSP1094) were acquired from the Marine Microbial Eukaryote Transcriptome Sequencing Project (Keeling et al., 2014). The trophic mode was predicted using HMMER3 version 3.3.2 (Mistry et al., 2013) and predictTrophicMode v1.0.0 (Burns et al., 2018).

### Prediction of Cyanobacterial Genes in the *B. bigelowii* Transcriptome

All contigs were searched for homology using DIAMOND version 0.9.14 (Buchfink et al., 2015) against the NCBI non-redundant protein database. The lowest common ancestor assignment was performed using MEGAN Community Edition version 6.18.39 (Huson et al., 2016) with two options (minimum score=20, percent to cover=60). The 215 transcripts, which were closely related to some genes of cyanobacteria, were used for phylogenetic analyses and searched for homology using blastp against the NCBI-refseq_protein database. If haptophytes were included in the top 50 blastp hits, the sequences were excluded. The top 50 hits were used as the database for phylogenetic analyses. Alignment was performed using MAFFT version 7.427 (Katoh and Toh, 2008) with the “linsi” option and trimmed using trimAl version 1.4.rev15 (Capella-Gutierrez et al., 2009) with the option “automated1.” ML trees were inferred using IQ-TREE version 1.6.12 (Nguyen et al., 2015) with 100 nonparametric bootstrap replicates.

## Results and Discussion

### The *C. parkeae* Stage of *B. bigelowii* and Its Endosymbiont

**Figure 1:**
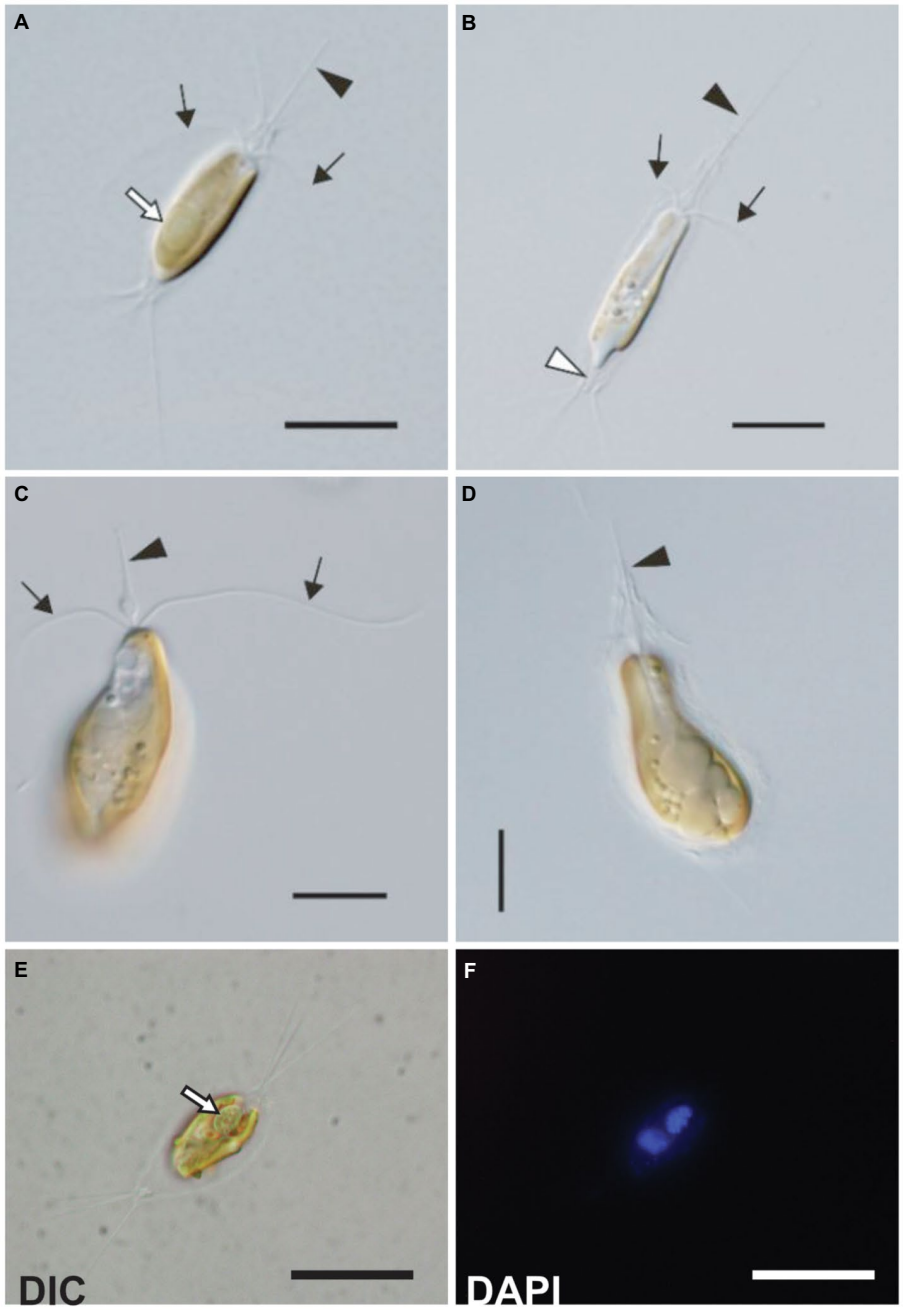
Light microscopy images of the *C. parkeae* stage of *B. bigelowii*. Two strains KC1-P2 **(A**,**B**,**E**, and **F)** and KC15-24 **(C** and **D)** are shown. Cells possessed a haptonema (black arrowheads), two flagella (black arrows), and a distinctive projecting structure at the posterior end (white arrowhead). **(A)** KC1-P2 with its endosymbiont. KC1-P2 possessed a single endosymbiont at the posterior of the cell (white arrow). **(B)** KC1-P2 without its endosymbiont. KC1-P2 lost its endosymbiont at the beginning of cultivation. **(C** and **D)** KC15-24 did not possess an endosymbiont. A swelling of the haptonema is visible at the base. **(E**,**F)** Fluorescence microscopy images of KC1-P2 stained using DAPI. Blue fluorescence of the endosymbiont shows the presence of DNA. Scale bars represent 10μm.

Strains MK90-06, KC1-P2, and KC15-24 were oval or pyriform, but their cell sizes were slightly different; strains MK90-06 and KC1-P2 were 15–22μm long and 5–9μm wide, and strain KC15-24 was 18–26μm long and 7–10μm wide (Figures 1A–D). Cells possessed two flagella of equal length and one haptonema with a basal swelling (Figure 1C). Coiling of the haptonema was not observed. Cells were covered with multi-layered oval scales of three types and 3–6 long spine-like scales at the anterior and posterior ends (Figures 1A–D; Supplementary Figures 1, 2). These morphological features were consistent with the original description of *C. parkeae* (Green and Leadbeater, 1972), synonymous with *B. bigelowii* (Hagino et al., 2013); therefore, we identified these strains as the *C. parkeae* stage of *B. bigelowii*. These results were consistent with the phylogenetic analyses using 18S rRNA and plastid 16S rRNA (Supplementary Figures 3, 4). Phylogenetic analysis showed that strains MK90-06 and KC1-P2 corresponded to genotype III of *B. bigelowii* and that KC15-24 corresponded to genotype IV (Supplementary Material).

**Figure 2:**
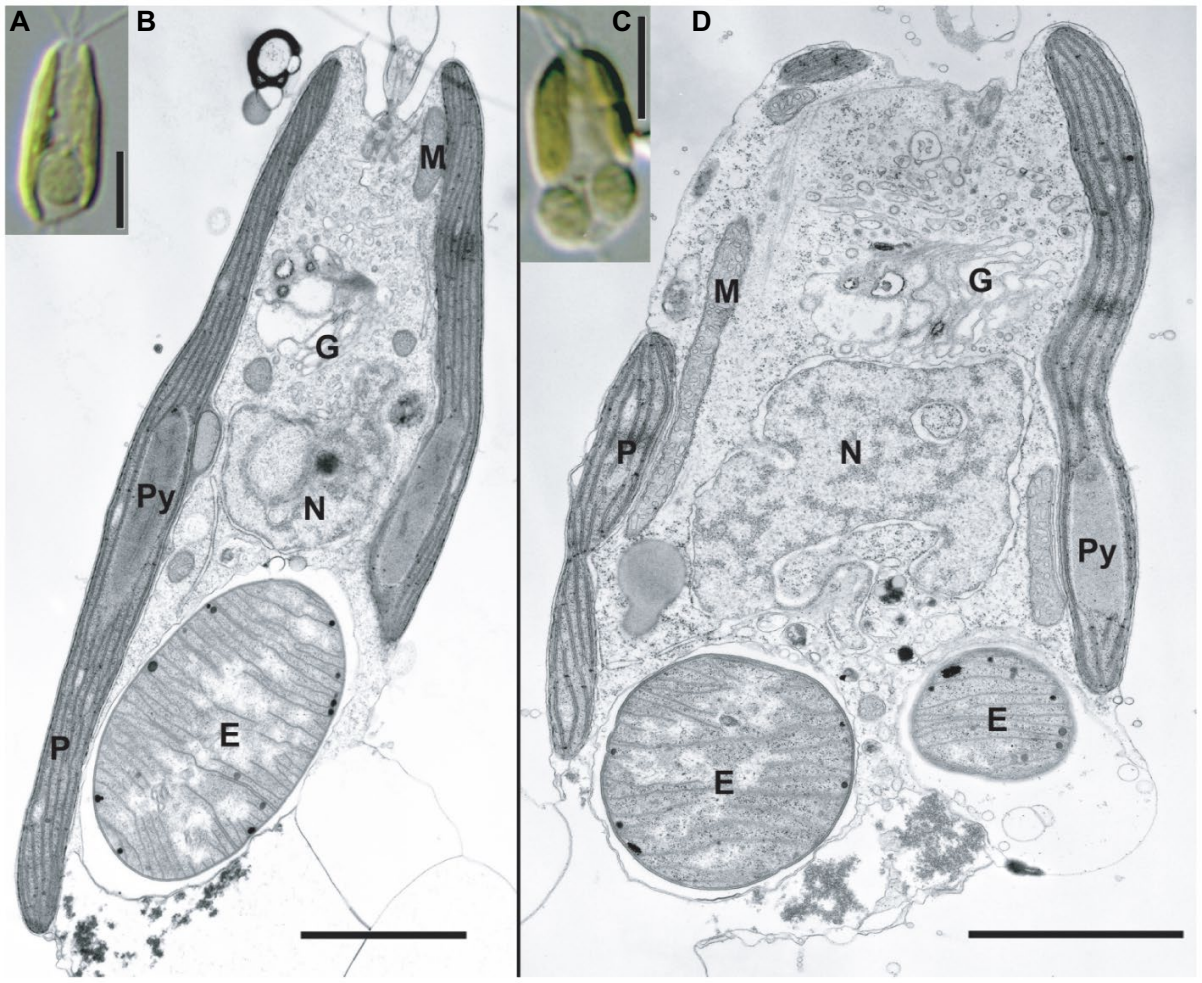
TEM images of the *C. parkeae* stage of *B. bigelowii*. Light microscopy images **(A**,**C)** and corresponding TEM images are shown **(B**,**D)** for strain MK90-06. Usually, one endosymbiont is found per cell in the posterior of the cell **(A**,**B)**; however, some of the cells had two endosymbionts per cell during cell division **(C**,**D)**. Scale bars represent 5μm in **(A** and **C)**, and 2μm in **(B** and **D)**. E, endosymbiont; G, Golgi apparatus; M, mitochondrion; N, nucleus; P, plastid; and Py, pyrenoid.

Strains KC1-P2 and MK90-06 possessed a spheroid structure between two plastids at the posterior of the cell (Figures 1A,E,F and 2A,B), although strain KC15-24 did not (Figures 1C,D). We stained KC1-P2 cells with DAPI, and blue fluorescence was observed in the spheroid structure (Figures 1E,F), indicating the presence of DNA in this structure. Interestingly, the spheroid structure of KC1-P2 was lost during cultivation (Figure 1B), suggesting that this structure is not essential for the growth of *B. bigelowii*, at least under our laboratory culture conditions. To observe the ultrastructure of this spheroid structure, we performed TEM on strain MK90-06 (Figures 2A–D; Supplementary Figure 5). The TEM observations indicated that this spheroid structure possessed cyanobacterium-like features: two membranes, a peptidoglycan layer, and lateral thylakoids, which are similar to those found in cyanobacteria, such as *Cyanothece* sp. ATCC 51142 (Liberton et al., 2011). This spheroid structure was surrounded by a single membrane, which was fused with a nuclear membrane, possibly originating from the food vacuole of *B. bigelowii* (Supplementary Figure 5).

*B. bigelowii* (in the coccolithophore stage) possesses a cyanobacterial endosymbiont with a similar structure (Hagino et al., 2013), suggesting that *B. bigelowii* can maintain the endosymbiont throughout its life cycle. Some MK90-06 cells contained two endosymbionts during the initial cell division, indicating that the endosymbiont could divide synchronously in the host cell and be vertically transferred to the offspring.

To elucidate the phylogenetic relationships of the *B. bigelowii* endosymbiont, we performed a phylogenetic analysis using the *nifH* genes including that of the genome of *B. bigelowii* endosymbiont (Supplementary Figure 6). Based on the *nifH* sequence, the endosymbiont of *B. bigelowii* was monophyletic with uncultured cyanobacteria, UCYN-A2, with moderate bootstrap support (*BP*=79). In the dataset, the *nifH* sequence of the endosymbiont of *B. bigelowii* was the most similar to that of the UCYN-A2 genome (JPSP01000000; Bombar et al., 2014) with 99.66% nucleotide similarity. These results suggest that UCYN-A2 is the endosymbiont of *B. bigelowii* genotype III as described in Thompson et al. (2014).

KC15-24 lacked an endosymbiont; however, it was unclear whether KC15-24 lacked an endosymbiont in nature, or whether its endosymbiont was lost at the beginning of preculture. Green and Leadbeater (1972) described this species without such a spheroid structure, although it could have been overlooked. Regardless, we speculate that *B. bigelowii* can discharge or digest the endosymbiont (discussed below) and the events might be relatively rare because the strain MK90-06 had been maintained with the endosymbiont over a year under our laboratory condition.

### Genomic Features of the *B. bigelowii* Endosymbiont

**Figure 3:**
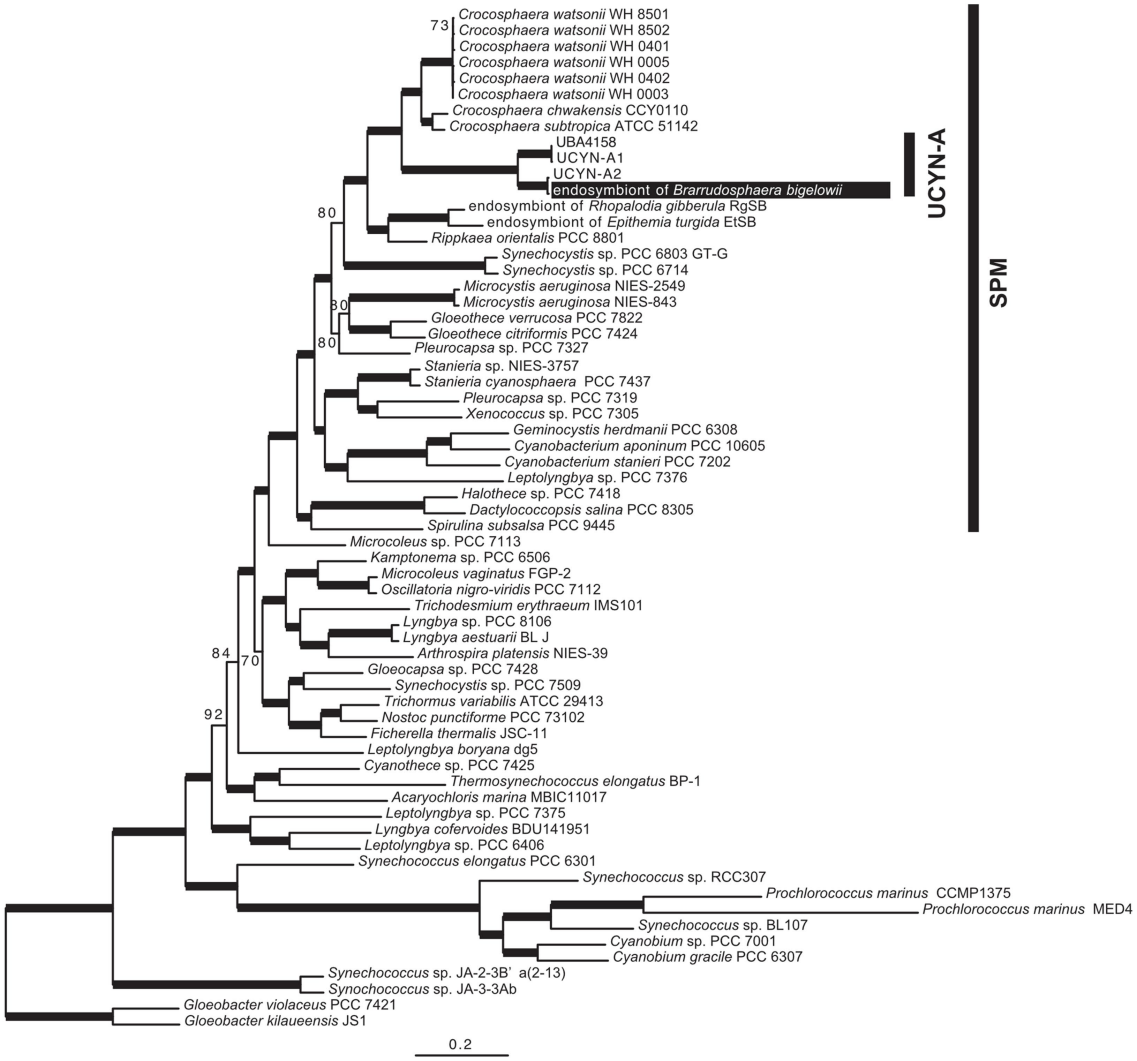
Phylogenetic tree based on the 160 proteins encoded in the genome of the *B. bigelowii* endosymbiont. The ML tree was inferred using 160 proteins of 64 cyanobacteria (46,587 amino acids). Bootstrap values (BP) are shown on nodes. Bold lines indicate a BP value of 100.

We sequenced the complete genome of an endosymbiont of an isolated single cell of *B. bigelowii* (Supplementary Figure 7). To the best of our knowledge, this is the first report in which the genome of a single or clonal UCYN-A cell was sequenced, in contrast to other studies in which population sequencing was employed (Tripp et al., 2010; Thompson et al., 2012; Bombar et al., 2014). Phylogenetic analysis using 160 orthologous proteins showed that the endosymbiont was closely related to UCYN-A2 and that these two genomes formed a monophyletic group with UCYN-A1 and UBA4158 (derived from metagenomic data; Parks et al., 2017) with robust bootstrap support (*BP*=100; Figure 3). The topology of this tree was consistent with that of the *nifH*-derived tree (Supplementary Figure 6).

The *B. bigelowii* endosymbiont genome comprised 1,496,411bp, which was slightly longer than that of UCYN-A1 and UCYN-A2 (Supplementary Table 4). The *B. bigelowii* endosymbiont genome encoded 1,188 coding sequences (CDSs), 37 tRNAs, and 6 rRNAs (Supplementary Table 4). Most of the CDSs of the *B. bigelowii* endosymbiont were shared with UCYN-A1 (1,137 CDSs, 95.7% of the total CDSs of the *B. bigelowii* endosymbiont) and UCYN-A2 (1,184 CDSs, 99.7% of the CDSs). As described in UCYN-A1 and UCYN-A2 (Tripp et al., 2010; Bombar et al., 2014), the endosymbiont possessed genes for nitrogen fixation and lacked genes for the TCA cycle, photosystem II, and Calvin cycle. In particular, the *B. bigelowii* endosymbiont and UCYN-A2 possessed the nearly same gene repertories. All of the CDSs of UCYN-A2 were found in the *B. bigelowii* endosymbiont genome, whereas the endosymbiont possessed only five unique CDSs, which were absent in UCYN-A2 (Supplementary Table 5). Of them, one CDS was probably located in an inter-contig region of the UCYN-A2 assembly, and the remaining four CDSs are likely to be pseudogenized in UCYN-A2 because of the presence of stop codons or flame shifts in these CDSs.

However, there were some unique genes that were absent or pseudogenized in either the *B. bigelowii* endosymbiont or UCYN-A1 (40 and 36 unique genes, respectively; Supplementary Tables 5 and 6). Most of these genes encoded hypothetical proteins, and thus, some of them might be misannotated. The *B. bigelowii* endosymbiont lacked two genes for transcriptional regulators, a GntR family transcriptional regulator, and a putative transcriptional regulator. Moreover, a gene encoding a putative transcriptional regulator was pseudogenized in both genomes, and the *B. bigelowii* endosymbiont possessed a pseudogene of the RpoD/SigA family RNA polymerase sigma factor, which was completely absent in UCYN-A1. These results suggest that the *B. bigelowii* endosymbiont and UCYN-A1 may lack some components of their transcriptional regulatory systems. A similar situation was observed during the organellar genome reduction of secondary endosymbiosis in plastid acquisition (Suzuki et al., 2016). However, previous studies showed that *nifH* of UCYN-A1 and UCYN-A2 had a diurnal transcriptional pattern, and the highest level of *nifH* expression was observed during the day (Church et al., 2005; Thompson et al., 2014). The expression of *nifH* is regulated by CnfR in the heterocyst-lacking cyanobacterium *Leptolyngbya boryana* (Tsujimoto et al., 2014), and the *B. bigelowii* endosymbiont (UCYN-A2) and UCYN-A1 possess homologs of *cnfR* (CPSB_00856 and UCYN_05600, respectively), suggesting that they do not lack the entire transcriptional regulatory system.

UCYN-A1 lacked genes for RecBCD [related to double-stranded DNA break repair (Cassier-Chauvat et al., 2016)], and the *B. bigelowii* endosymbiont also lacked these genes. Only the *B. bigelowii* endosymbiont (UCYN-A2) lacked genes for DNA repair, *recO*, and *xerD*. We could not find these intact genes even in the intergenic regions of the *B. bigelowii* endosymbiont genome using blastx search. These genes are broadly found in various cyanobacteria, including UCYN-A1 (Cassier-Chauvat et al., 2016). RecO repairs single-stranded DNA nicks. Together with RecF and RecR, the RecFOR complex recognizes gaps, which are enlarged by RecQ and RecJ. Deletion of *recO* results in the failure of DNA replication recovery in *E. coli* (Chow and Courcelle, 2004). This information suggests that the *B. bigelowii* endosymbiont lacks the RecF pathway for repairing single-stranded DNA nicks. This is supported by the absence of the *xerD* gene, which encodes a site-specific recombinase that resolves DNA dimers into monomers during cell division (Castillo et al., 2017). Because bacterial transcripts encoding RecO and XerD could not be found in our RNA-Seq analysis of *B. bigelowii*, these proteins may not be transferred from the host cell to the endosymbiont. The nuclear genome sequencing of *B. bigelowii* is required to elucidate the absence of these genes.

To investigate genome rearrangement among UCYN-A1, UCYN-A2, and the *B. bigelowii* endosymbiont, we performed the progressiveMauve analysis (Darling et al., 2010). We found a single inverted region (approximately 16kb) of the *B. bigelowii* endosymbiont using UCYN-A1 as a reference (Supplementary Figure 8). This inverted region was composed of 16 and 15 CDSs (*ribH* to *glycosyl transferase*) in UCYN-A1 and the *B. bigelowii* endosymbiont, respectively. The ends of the inverted region occurred in intergenic regions (CPSB_00460–CPSB_00461 and *glycosyl transferase*–*frr*) and did not disturb the genetic structure. We reordered the contigs of UCYN-A2 using the *B. bigelowii* endosymbiont genome as a reference (Supplementary Figure 8), and they showed the completely same order of genes without an inversion, illuminating the close phylogenetic relationship of these genomes (Figure 3).

### Nitrogen Metabolism in *B. bigelowii*

To elucidate the genetic adaptations of the host to the nitrogen-fixing endosymbiont, we performed RNA-Seq on endosymbiont-free *B. bigelowii* (strain KC1-P2). In the RNA-Seq analysis, we could not detect any contamination of the endosymbiont transcripts, such as 16S rRNA, although nuclear 18S rRNA and plastid 16S rRNA were detected (Supplementary Table 7). This result indicates that this strain of *B. bigelowii* completely lacks its endosymbiont.

**Figure 4:**
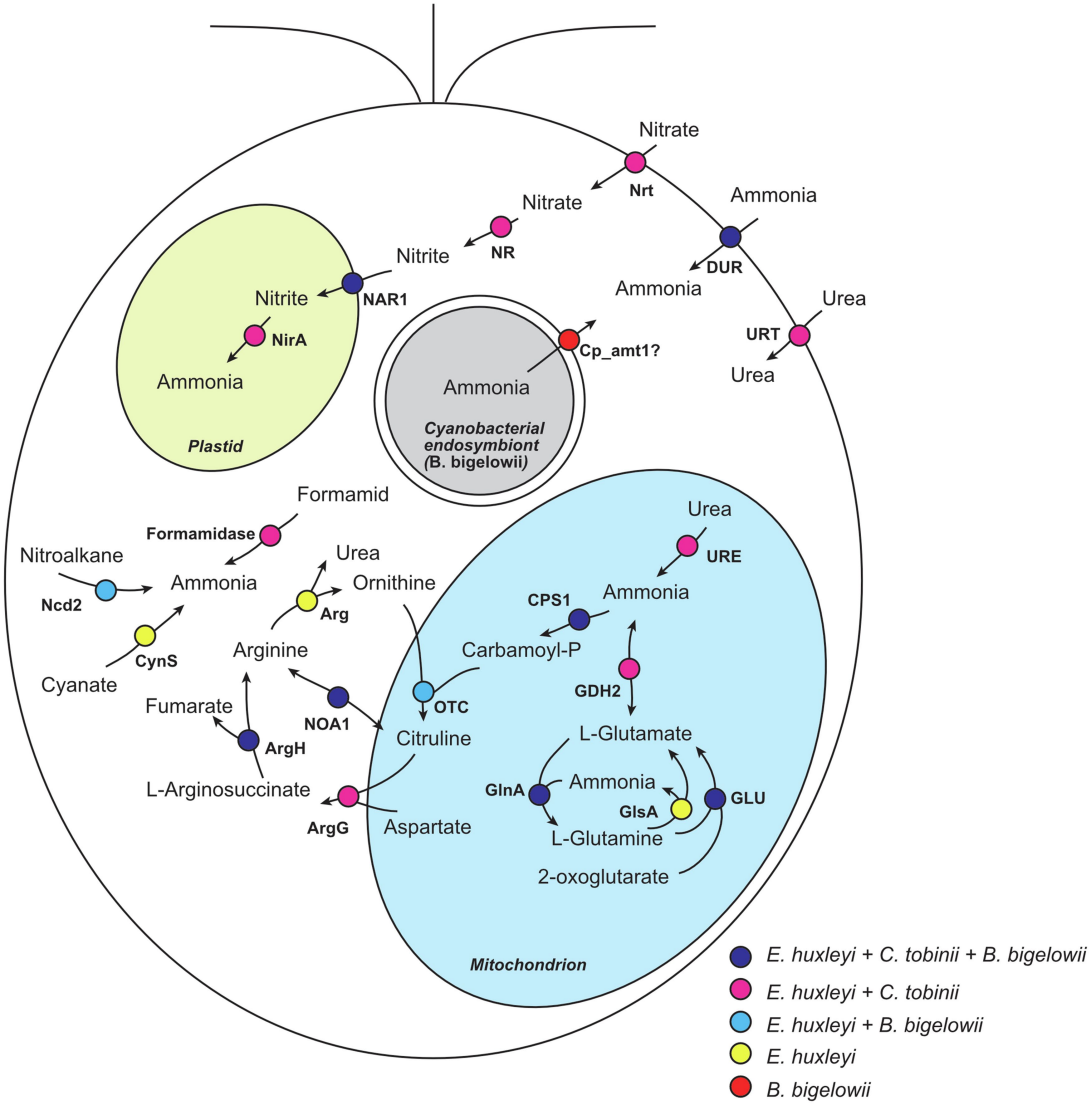
Nitrogen metabolism in haptophytes. Nitrogen metabolism was predicted based on the genome of *E. huxleyi*. Proteins encoded in the genomes of *E. huxleyi* and *C. tobinii*, and the transcriptome of *B. bigelowii* is shown. Circles with different colors represent proteins shared among different species. Nrt, nitrate transporter; NR, nitrate reductase; NAR1, formate/nitrite transporter; NirA, ferredoxin-nitrite reductase; Ncd2, nitronate monooxygenase; CynS, cyanate lyase; DUR, ammonia transporter; URT, urea transporter; URE, urease; GDH2, glutamate dehydrogenase; GLU, glutamate synthase; GlsA, glutaminase; GlnA, glutamine synthetase; CPS1, carbamoyl-phosphate synthase; OTC, ornithine carbamoyltransferase; ArgG, argininosuccinate synthase; ArgH, argininosuccinate lyase; NOA1, nitric-oxide synthase; and Arg, arginase.

Interestingly, we found that some transcripts related to nitrogen uptake, storage, and reuse were absent from the *B. bigelowii* transcriptome. We compared the genomes of two haptophytes that do not fix nitrogen, *Emiliania huxleyi* (marine species; Read et al., 2013) and *Chrysochromulina tobinii* (freshwater species; Hovde et al., 2015), to the transcriptome of *B. bigelowii* (Figure 4; Supplementary Table 8). The *E. huxleyi* genome possesses genes for proteins capable of producing ammonia from various nitrogen sources: nitrate, nitrite, formamide, glutamine, glutamate, cyanate, and nitroalkane. The *C. tobinii* genome lacked genes for nitronate monooxygenase, cyanate lyase, and arginase, suggesting that *C. tobinii* cannot produce ammonia from nitroalkane and cyanate, or store nitrogen as urea in cells. This is likely because its habitat is freshwater, which is a more nutrient-rich environment than the ocean. In contrast, interestingly, the *B. bigelowii* transcriptome lacked transcripts for nitrate transporters, nitrate reductase, nitrite reductase, formamidase, glutamate dehydrogenase, glutaminase, cyanate lyase, arginase, and urease. We could not detect these transcripts at the raw read level (Supplementary Table 8). These deficiencies suggest that under these conditions, *B. bigelowii* cannot use most nitrogen sources, such as nitrate, nitrite, formamide, glutamate, glutamine, cyanate, and urea, or store excess nitrogen as urea in cells. The RNA-Seq data contained sufficient amount of reads (>27 Gbp), and BUSCO analysis (Simão et al., 2015) showed relatively high gene completeness based on the eukaryote dataset (81.5% complete BUSCOs and 5.9% fragmented BUSCOs). Moreover, the RNA was extracted from cells in nitrogen-rich/-poor media and under a light–dark phase (Supplementary Table 3). For some algae and plants, the presence of nitrate induces the expression of genes encoding nitrate transporters (Clarkson and Lüttge, 1991; Navarro et al., 1996; Crawford and Glass, 1998; Daniel-Vedele et al., 1998; Suzuki et al., 2019); however, we could not detect the transcript in our RNA-seq analyses with nitrate. Together with the essential function of these genes in nitrogen metabolism, it is possible that these genes are absent or pseudogenized in the genome.

In contrast, *B. bigelowii* expressed some genes for the ammonia transporter with higher expression values in the total transcripts (Figure 4; Supplementary Table 9). The genome of the *B. bigelowii* endosymbiont possessed an intact *nif* gene cluster (*nifHDKBEN*), suggesting that it can fix nitrogen and produce ammonia. A symbiotic exchange of fixed nitrogen, produced by UCYN-A, and for fixed carbon, produced by its haptophyte host, were observed using HISH-SIMS methodology (Thompson et al., 2012; Martínez-Pérez et al., 2016; Mills et al., 2020). We suggest that a similar exchange may occur between *B. bigelowii* and its endosymbiont. These findings suggest that *B. bigelowii* may have lost or ceased expressing most of its “unnecessary” nitrogen acquisition system because of the presence of the nitrogen-fixing endosymbiont. This bias in the usability of nitrogen resources of *B. bigelowii* can explain the previous report that *B. bigelowii* relies on nitrogen generated by the endosymbiont even in nitrogen-rich environments (Mills et al., 2020).

However, we can maintain endosymbiont-free *B. bigelowii* strains in ESM medium with/without nitrate, which does not contain ammonia, suggesting the presence of different supply sources of ammonia to *B. bigelowii*. Our RNA-seq analyses showed that the strain was maintained with some bacteria related to *Mesorhizobium*, *Kyptococcus*, and an unidentified Actinobacterium (Supplementary Table 7 and Supplementary Figure 9). In particular, the *Mesorhizobium* genus contains symbiotic species capable of nitrogen fixation (Jarvis et al., 1997) and nitrate reduction (Yang et al., 2020), and has been isolated from marine environments (Hagström et al., 2000; Yang et al., 2020). Therefore, this species can provide fixed ammonia to *B. bigelowii* by diffusion through the medium or phagocytosis (discussed below). All of these bacteria can produce ammonia from organic nitrogen, such as amino acids, and thus, these kinds of bacteria may provide ammonia to *B. bigelowii* under organic nitrogen-rich environments, for example, high dissolved organic matter. This interspecies interaction might increase the likelihood of endosymbiont loss from *B. bigelowii* because the endosymbiont is not the sole supplier of ammonia.

### Putative Nutrient Transport Mechanisms

To elucidate the mechanism of nutrient transport between *B. bigelowii* and its endosymbiont or other extracellular organisms, we searched for sugar uptake transporters in the endosymbiont, and sugar efflux and ammonium uptake systems in *B. bigelowii*. The endosymbiont possessed ABC transporter genes for sugar uptake (CPSB_00277, CPSB_00343, CPSB_00347, and CPSB_00506; Supplementary Table 10), which were shared among various cyanobacteria, suggesting that it imports sugars from its surroundings.

To exchange nutrients with the endosymbiont, the host needs an ammonia transporter to receive fixed ammonia, and a sugar efflux transporter to export fixed carbon. We found 15 transcripts for putative ammonium transporters in our RNA-Seq data. Although most of the transcripts were shared with *E. huxleyi*, one transcript (Cp_amt1; KC1-P2_N3_k49_locus_8953_Transcript_1_1) was absent in the genomes of *E. huxleyi* and *C. tobinii*. Phylogenetic analysis showed that this transcript in *B. bigelowii* was closely related to that of a colonial haptophyte, *Phaeocystis globosa*, and both species were in a branch within the SAR supergroup (Supplementary Figure 10). These results suggest that the Cp_amt1 gene was acquired from SAR species *via* horizontal gene transfer. *Phaeocystis* occurs as an endosymbiont in acantharean species and is maintained without digestion by the host (Mars Brisbin et al., 2018). A related ammonium transporter was also found in the genome of *Symbiodinium microadriaticum*, which is a symbiont of corals. In the *S. microadriaticum* genome, protein families of ammonium transporters are extensively expanded and thought to be a key element for endosymbiosis (Aranda et al., 2016). These findings imply that this type of ammonium transporter might be utilized to exchange nitrogen compounds between the host and symbiont.

Some haptophytes are known to perform phagocytosis to uptake other organisms as prey (Kawachi et al., 1991; Jones et al., 1995; Tillmann, 1998). Therefore, we predicted trophic modes of our *B. bigelowii* strain using whole transcripts, based on the model by Burns et al. (2018). Together with *B. bigelowii*, we predicted trophic modes of a phagotrophic haptophyte, *Haptolina brevifila* (Jones et al., 1995). Both species showed high phagocyte prediction scores with 0.98 and 0.95 for *H. brevifila* and *B. bigelowii*, respectively (Supplementary Table 11). Although we could not find intake of other organisms by our electron microscopic observation, this prediction result strongly suggests potential phagocytosis of *B. bigelowii*. *B. bigelowii* might ingest its endosymbiont by the phagocytosis system. After loss of the endosymbiont, *B. bigelowii* might acquire nitrogen compounds *via* phagocytosis as well as direct absorption of ammonia.

Bacterial and fungal plant pathogens control mRNA levels of the host genes for SWEET, which is a sugar efflux transporter, to acquire glucose from plant cells (Chen et al., 2010). We found six transcripts that belong to the SWEET protein family (PF03083) in our RNA-Seq data. Four of these had phylogenetic affinity with other algae, including *E. huxleyi*. The two remaining transcripts (Cp_SemiSWEET1_1; KC1-P2-N_k25_Locus_2235_Transcript_6_1 and Cp_SemiSWEET1_2; KC1-P2-N_k37_Locus_3337_Transcript_3_1) had three transmembrane helices, which indicated that these encoded SemiSWEET proteins. These transcripts were closely related to a gene in *C. tobinii* (KOO25604.1), and the outgroups were composed of various bacteria, but no eukaryotes (Supplementary Figure 11), suggesting that these *B. bigelowii* genes were acquired in a common ancestor of *B. bigelowii* and *C. tobinii* from a bacterium *via* horizontal gene transfer. Although *C. tobinii* does not possess an endosymbiont, these transcripts of *B. bigelowii* possessed a long extension (corresponding to ~160 amino acids at N-termini), which was absent in *C. tobinii* and bacteria, implying functional variation among the different species. Therefore, genes for the ammonium transporter (Cp_amt1) and sugar transporters (Cp_SemiSWEET1_1 and Cp_SemiSWEET1_2) are good candidates that may play roles in nitrogen–carbon exchange between symbionts and the host. To elucidate the exact functions of proteins encoded by these genes, more culture-based experiments, e.g., to determine the subcellular localization, are required.

### Horizontal Gene Transfer From Cyanobacteria to *B. bigelowii*

During the acquisition of endosymbionts, such as in early plastid evolution, some endosymbiont genes are transferred into the host genome [endosymbiotic gene transfer (EGT); Ponce-Toledo et al., 2019]. To examine the possibility of EGT between *B. bigelowii* and its endosymbiont, we searched for “cyanobacterial” transcripts in the RNA-Seq data from *B. bigelowii*. Initial screening based on a homology search showed 215 transcripts were closely related to some genes of cyanobacteria. For these candidates, we performed phylogenetic analyses to elucidate their phylogenetic origins. Although phylogenetic positions of most of the candidates were inferred with long branch and low support values, we found nine “cyanobacterial” transcripts for glutathione S-transferase, lytic transglycosylase, and *N*-acetylmuramoyl-l-alanine amidase, all of which were absent in the endosymbiont genome. However, their phylogenetic positions were different from those of the endosymbiont, which was located in the SPM group (Figure 3; Bombar et al., 2014). These results suggest that these genes were acquired from a cyanobacterium, independent of the endosymbiotic event.

Transcripts for glutathione S-transferase (KC1-P2_N1_k49_Locus_14859_Transcript_1_1 and KC1-P2-N_CL7609Contig1_1) were monophyletic with filamentous or unicellular cyanobacteria capable of nitrogen fixation (*Crocosphaera*, *Microcoleus*, *Mastigocoleus*, and *Nostoc*; Supplementary Figure 12), suggesting that these transcripts were related to nitrogen fixation. Glutathione S-transferase plays a role in protecting nitrogenase from oxygen in root nodules of land plants (Dalton et al., 2009). Glutathione S-transferase is also used to detoxify cyanotoxins and liposaccharides generated by cyanobacteria in some eukaryotes, such as *Daphnia* (Ferrão-Filho and Kozlowsky-Suzuki, 2011). Although the *B. bigelowii* endosymbiont did not possess genes for the synthesis of most secondary metabolites, its free-living relative, *Crocosphaera watsonii* strain WH 8501 possessed genes for the synthesis of various kinds of secondary metabolites (bacteriocin, nematophin, minutissamides, puwainaphycins, anabaenopeptin, aeruginosides, and aranazole; Supplementary Table 13), suggesting that *B. bigelowii* may have needed to detoxify secondary metabolites during the early evolution of endosymbiosis. In any case, this cyanobacterial glutathione S-transferase might contribute to the success and maintenance of this endosymbiosis.

The transcripts of lytic transglycosylase (KC1-P2_N3_k25_Locus_1918_Transcript_1_1 and KC1-P2_N3_k55_Locus_6120_Transcript_1_1) and *N*-acetylmuramoyl-L-alanine amidase (KC1-P2-N_CL2Contig44_1, KC1-P2-N_CL307Contig1_1, KC1-P2_N1_k49_Locus_4594_Transcript_1_1, KC1-P2_N3_k25_Locus_11130_Transcript_1_1, and KC1-P2_N3_k31_Locus_8635_Transcript_1_1) were monophyletic with *Cyanobium*, *Prochlorococcus*, and *Synechococcus* (SynPro clade; Supplementary Figures 13, 14). Both of these proteins are related to peptidoglycan metabolism and cell division in cyanobacteria. Lytic transglycosylase digests peptidoglycan between *N*-acetylglucosamine (GlcNAc) and *N*-acetylmuramic acid (MurNAc), creating space for cell growth (Scheurwater et al., 2008). This protein is used to separate daughter cells during bacterial cell division (Heidrich et al., 2002). *N*-acetylmuramoyl-L-alanine amidase degrades the 1,6-anhydromuramyl moiety, generated by lytic transglycosidase, to MurNAc to recycle peptidoglycan. These proteins were not found in the other haptophyte genomes in PhycoCosm (Grigoriev et al., 2021). Although it remains unknown that these proteins have been acquired prior or posterior to the endosymbiosis of the cyanobacterium, these proteins might be related to maintaining the endosymbiont.

### Fate and Ecological Implications of the *B. bigelowii* Endosymbiont

Previous studies have shown that *B. bigelowii* and its endosymbionts have several ecotypes and genotypes (Hagino et al., 2009; Thompson et al., 2012; Farnelid et al., 2016). UCYN-A2 (the *B. bigelowii* endosymbiont) is considered to be a specific form adapted to the coastal environment, in contrast to UCYN-A1, which is adapted for the open ocean (Thompson et al., 2014). In this study, we compared their genomes and found that only the *B. bigelowii* endosymbiont (UCYN-A2) lacked the RecF pathway for DNA repair, as well as the RecBCD pathway. The loss of DNA repair mechanisms can destabilize the endosymbiont genome. Moreover, we showed that *B. bigelowii* can lose its endosymbiont during cultivation and grow under an external nitrogen supply. *B. bigelowii* can likely take up ammonia produced by free-living bacteria, implying that the nitrogen–carbon exchange between *B. bigelowii* and its endosymbiont is not obligate. We also found that *B. bigelowii* did not express genes originating from its endosymbiont, suggesting that their genetic connection is looser than other early endosymbiotic processes. For example, *Paulinella chromatophora*, which corresponds to an early stage of plastid acquisition, possesses many genes that originate from the endosymbiont (Nowack et al., 2016). Based on these results, we speculate that the *B. bigelowii* endosymbiont may be an early stage of endosymbiosis before it is established as an organelle and disappear under ammonia-rich conditions, in contrast to UCYN-A1. In the future, nuclear genome sequencing of *B. bigelowii* provides robust evidence for our results.

## Data Availability Statement

The datasets presented in this study can be found in online repositories. The names of the repository/repositories and accession number(s) can be found at repository: DDBJ. The accessions are follows: DRA011127, DRA011134, AP024987, LC595680, LC595681, and LC595682.

## Author Contributions

SS, MK, II, and K-II designed the research. SS, MK, CT, AN, and KH performed the research. SS analyzed the data. SS, MK, and KH wrote the paper. All authors contributed to the article and approved the submitted version.

## Funding

This study was funded by the JSPS KAKENHI (grant nos. 14J00572 and 19K15904 to SS). SS received a grant from the Institute for Fermentation, Osaka, Japan (G-2019-1-043). This work was partially supported by the National BioResource Project for Algae under grant no. 17km0210116j0001, which is funded by the Japan Agency for Medical Research and Development (AMED).

## Conflict of Interest

The authors declare that the research was conducted in the absence of any commercial or financial relationships that could be construed as a potential conflict of interest.

## Publisher’s Note

All claims expressed in this article are solely those of the authors and do not necessarily represent those of their affiliated organizations, or those of the publisher, the editors and the reviewers. Any product that may be evaluated in this article, or claim that may be made by its manufacturer, is not guaranteed or endorsed by the publisher.

## Footnotes

^1^ https://github.com/TransDecoder/TransDecoder/releases/tag/TransDecoder-v5.5.0
