# Supplementary material for: Unstable Relationship Between Braarudosphaera bigelowii (= Chrysochromulina parkeae) and Its Nitrogen-Fixing Endosymbiont
Source: Front Plant Sci. 2021 Dec 3;12:749895. doi: 10.3389/fpls.2021.749895 (PMC8679911; doi:10.3389/fpls.2021.749895)
Supplement: Supplementary file 1 [file Data_Sheet_1.docx]

Supplementary Material

# Supplementary Text

**Phylogenetic relationships of *B. bigelowii* strains.** To infer the phylogenetic relationships of the three strains, we performed phylogenetic analyses using genes for 18S rRNA and plastid 16S rRNA (Supplementary Figures. 2–4). The 18S rRNA and plastid 16S rRNA trees showed monophyly of *C. parkeae* and *B. bigelowii* with robust support values (BP = 92 and 100, respectively). In particular, the 18S rRNA sequences of KC1-P2 and MK90-06 were nearly identical to those of *B. bigelowii* genotype III (AB478413). In contrast, KC15-24 possessed the same 18S rRNA sequence as genotype IV (AB478413). *C. parkeae* and *B. bigelowii* were included in the order Prymnesiales with a low support value (BP = 56) in the 18S rRNA tree. In contrast, they were included in the Coccolithophore clade with a robust support value (BP = 96) in the plastid 16S rRNA tree. These results clearly show that every *B. bigelowii* genotype has a *C. parkeae* stage in its life cycle, and KC1-P2, MK90-06, and KC15-24 correspond to different genotypes of *B. bigelowii* (genotypes III and IV, respectively).

**Ultrastructures of *B. bigelowii* strains.** The TEM observations indicated that *B. bigelowii* has two additional unique structures near the endosymbiont: a projecting structure at the posterior end of the cell (black arrow in Supplementary Figures 2 and 5C) and electron-dense structures sandwiching the endosymbiont (Supplementary Figure 5D). The former is a structure for binding the spine scales, and the latter is probably a secondary metabolite or waste product derived from the endosymbiont.

# Supplementary Materials and Methods

**Transmission Electron Microscopy** To observe the mounted whole cells using TEM, cells (strain KC15-24) were collected by gentle centrifugation (2,000 × g, 3 min), and cells were collapsed by the addition of distilled water. Cell suspensions were mounted on formvar-coated grids with a 150-µm mesh, and the copper grids were stained with 2% (w/v) uranyl acetate for 2 min and washed twice with distilled water. Cells were observed using a Hitachi H-7650 electron microscope (Hitachi High-Technologies, Tokyo, Japan) equipped with a Veleta TEM CCD camera (Olympus, Tokyo, Japan). A detailed method for strain MK90-6 was described by Kawachi et al. (Kawachi et al., 1991).

**Molecular phylogenetic analyses using 18S rRNA, plastid 16S rRNA, and *nifH*** DNA was extracted from two strains, KC1-P2 and KC15-24, using the DNeasy Plant Mini kit (Qiagen, Hilden, Germany) following the manufacturer’s protocol. PCR was performed with KOD FX Neo polymerase (Toyobo, Osaka, Japan) using SR1 and SR12 primers for 18S rRNA (Nakayama et al., 1998). Primers specific for *B. bigelowii* plastid 16S rRNA were designed with the following sequences: cpsb_16S_F, 5- AGGATGAACGCTGGCGGTATGCC-3; cpsb_16S_R, 5-GTGATCCAGCCACACCTTCCGG-3′. Amplified DNA was visualized by electrophoresis, purified using the QIAquick Gel Extraction kit (Qiagen), and ligated into the pGEM-T easy vector (Promega, Madison, WI). The plasmids were sequenced with an ABI 3130 sequencer (Applied Biosystems, Foster City, CA, USA) using the BigDye version 3.1 kit (Applied Biosystems). The 18S rRNA sequence of strain MK90-06 was downloaded from GenBank (AM490994.1). The 18S rRNA sequences of KC1-P2 and KC15-24 were deposited in the DDBJ as accession nos. LC595680, and LC595681, respectively. The plastid 16S rRNA sequence of KC15-24 was deposited in DDBJ (accession no. LC595682). The nucleotide sequence for *nifH* was extracted from the genome of the *B. bigelowii* endosymbiont.

For phylogenetic analyses, datasets were generated for 18S rRNA, plastid 16S rRNA, and *nifH*, which comprised 89, 45, and 33 operational taxonomic units (OTUs), respectively. The dataset for 18S rRNA was essentially based on Edvardsen et al. ( 2011), and certain sequences from haptophytes found by BLASTN search were added. The sequences were aligned using MAFFT version 7.427 (Katoh and Toh, 2008), and trimmed using trimAl version 1.4.rev15 (Capella-Gutierrez et al., 2009). ML trees were analyzed using IQ-TREE version 1.6.12 (Nguyen et al., 2015). Non-parametric bootstrap analyses were repeated 100 times.

**Molecular phylogenetic analysis of *Mesorhizobium* sp.** rRNA sequences was performed using RNA-Seq contigs assembled using DRAP. The 16S and 18S rRNA sequences were predicted using SSU-ALIGN 0.1.1 (Nawrocki, 2009). Predicted rRNA sequences were confirmed by performing a BLASTN search against the NCBI NR database. Chimera regions were identified using DECIPHER 2.16.1 (Wright, 2016), and these regions were manually removed. Alignment was performed using MAFFT version 7.427 (Katoh and Toh, 2008) with the ‘linsi option’ and manually trimmed using MEGA 7 (Kumar et al., 2016). ML trees were inferred using IQ-TREE (Nguyen et al., 2015) with 100 nonparametric bootstrap replicates.

# Supplementary References

Capella-Gutierrez, S., Silla-Martinez, J. M., and Gabaldon, T. (2009). trimAl: a tool for automated alignment trimming in large-scale phylogenetic analyses. *Bioinformatics* 25, 1972–1973. doi:10.1093/bioinformatics/btp348.

Edvardsen, B., Eikrem, W., Throndsen, J., Sáez, A. G., Probert, I., and Medlin, L. K. (2011). Ribosomal DNA phylogenies and a morphological revision provide the basis for a revised taxonomy of the Prymnesiales (Haptophyta). *Eur. J. Phycol.* 46, 202–228. doi:10.1080/09670262.2011.594095.

Katoh, K., and Toh, H. (2008). Recent developments in the MAFFT multiple sequence alignment program. *Brief. Bioinform.* 9, 286–98. doi:10.1093/bib/bbn013.

Kawachi, M., Inouye, I., Maeda, O., and Chihara, M. (1991). The haptonema as a food-capturing device: observations on Chrysochromulina hirta (Prymnesiophyceae). *Phycologia* 30, 563–573. doi:10.2216/i0031-8884-30-6-563.1.

Kumar, S., Stecher, G., and Tamura, K. (2016). MEGA7: Molecular Evolutionary Genetics Analysis Version 7.0 for Bigger Datasets. *Mol. Biol. Evol.* 33, 1870–1874. doi:10.1093/molbev/msw054.

Nakayama, T., Marin, B., Kranz, H. D., Surek, B., Huss, V. A. R., Inouye, I., et al. (1998). The basal position of scaly green flagellates among the green algae (Chlorophyta) is revealed by analyses of nuclear-encoded SSU rRNA sequences. *Protist* 149, 367–380. doi:10.1016/S1434-4610(98)70043-4.

Nawrocki, E. (2009). Structural RNA Homology Search and Alignment using Covariance Models. *Washingt. Univ. Saint Louis, Sch. Med.* doi:10.1017/CBO9781107415324.004.

Nguyen, L.-T., Schmidt, H. A., von Haeseler, A., and Minh, B. Q. (2015). IQ-TREE: a fast and effective stochastic algorithm for estimating maximum-likelihood phylogenies. *Mol. Biol. Evol.* 32, 268–274. doi:10.1093/molbev/msu300.

Wright, E. S. (2016). Using DECIPHER v2.0 to Analyze Big Biological Sequence Data in R. *R J.* 8, 352. doi:10.32614/RJ-2016-025.

# Supplementary Figures and Tables

**Supplementary Figure 1.** **Whole-mount images of scales of KC1-P2.** Cells had rimmed plate scales (A, B), medium-sized plate scales (C, D), small plate scales (E), or long spines (F). The outsides (A, C, E) and insides (B, D) of scales are shown. These forms are consistent with the original description of *C. parkeae*. Scale bars represent 1 µm.

**Supplementary Figure 2.** **Whole-mount images of MK90-06 cells.** MK90-06 cells were covered with different kinds of scales. Rimmed plate scales (A), medium-sized plate scales (B), small plate scales (C), and long spines (D) are shown. These forms are consistent with the original description of *C. parkeae*. Cells possessed a projecting structure at the posterior end (black arrow). Scale bars represent 2 µm.

**Supplementary Figure 3.** **Phylogenetic tree of 18S rRNA.** The ML tree was inferred using 1,715 nucleotides from 89 OTUs with a TIM2e+I+G4 substitution model. Bootstrap supports (BP) are shown on each node. BP values <50 are not shown. Bold lines represent a BP value of 100. Red OTUs represent *B. bigelowii* strains.

**Supplementary Figure 4. Phylogenetic tree of plastid 16S rRNA.** The ML tree was inferred using 697 nucleotides from 45 OTUs with a K3Pu+F+I+G4 substitution model. Bootstrap supports (BP) are shown on each node. BP values <50 are not shown. Bold lines represent a BP value of 100. Red OTUs represent *B. bigelowii* strains.

**Supplementary Figure 5.** **TEM images of strain MK90-06 and its endosymbiont.** TEM was performed on strain MK90-06. The *C. parkeae* stage of *B. bigelowii* possessed an endosymbiont. (A) Endosymbiont in the host cell. The endosymbiont was surrounded by a single host membrane (white arrow). The single membrane was fused with a nuclear membrane (gray arrow). (B) Membrane structure of the endosymbiont. The endosymbiont possessed two membranes (black and white arrowheads), a peptidoglycan layer (white double arrowhead), and lateral thylakoids (black double arrowhead). (C) Cells possessed a projecting structure at the posterior end (black arrow). (D) The endosymbiont was sandwiched between electron-dense structures. E, endosymbiont; Es, electron-dense structure. Scale bars represent 1 µm (A, C, and D) or 100 nm (B).

**Supplementary Figure 6. Phylogenetic tree of *nifH*.** The ML tree was inferred using 864 nucleotides (524 constant nucleotides) from 33 OTUs with a TIM2e+G4 substitution model. Bootstrap supports (BP) are shown on each node. BP values <50 are not shown. Bold lines represent a BP of 100. The red OTU represents the *B. bigelowii* endosymbiont.

**
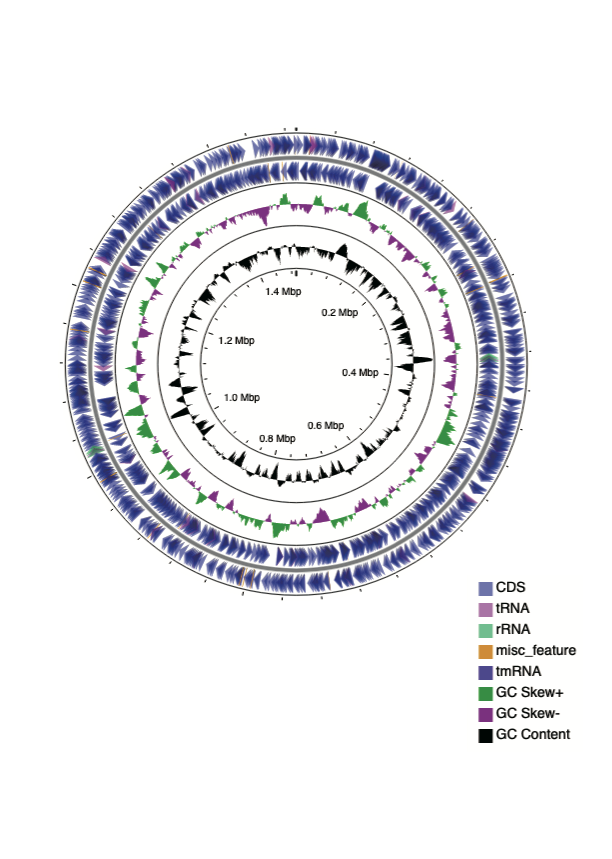
**

**Supplementary Figure 7. Genomic map of the *B. bigelowii* endosymbiont.** The two outermost circles are features (CDSs, tRNAs, rRNAs, tmRNAs, and miscellaneous features) encoded on forward and reverse strands of the genome. Counting from the outermost circle, the third circle is GC skew, and the fourth circle is GC content.

**Supplementary Figure 8. Comparison of genomic organization between UCYN-A1 and the *B. bigelowii* endosymbiont.** (A) Locally collinear blocks between UCYN-A1 and the *B. bigelowii* endosymbiont by the progressiveMauve analysis. (B) Locally collinear blocks between UCYN-A2 and the *B. bigelowii* endosymbiont by the progressiveMauve analysis. Corresponding locally collinear blocks are linked by lines. (C) Detailed map of a genomic inversion between UCYN-A1 and the *B. bigelowii* endosymbiont. The inverted region is filled in black.

**Supplementary Figure 9. Phylogenetic tree of 16S rRNA from a co-cultivated bacterium.** The ML tree was inferred using 1,482 nucleotides from 14 OTUs with a TPM2u+F+I substitution model. Bootstrap supports (BP) are shown on each node. BP values <50 are not shown.

**Supplementary Figure 10. Phylogenetic tree of Cp_amt1.** The ML tree was inferred using 289 amino acids derived from 22 OTUs with a LG+F+G4 substitution model. Bootstrap supports (BP) are shown on each node.

**Supplementary Figure 11. Phylogenetic tree of Cp_SemiSWEET1.** The ML tree was inferred using 78 nucleotides from 19 OTUs with an mtZOA+G4 substitution model. Bootstrap supports (BP) are shown on each node.

**Supplementary Figure 12. Phylogenetic tree of glutathione S-transferase.** The ML tree was inferred using 119 amino acids derived from 52 OTUs with an LG+I+G4 substitution model. Bootstrap supports (BP) are shown on each node.

**Supplementary Figure 13. Phylogenetic tree of lytic transglycosylase.** The ML tree was inferred using 223 amino acids derived from 102 OTUs with an LG+F+I+G4 substitution model. Bootstrap supports (BP) are shown on each node.

**Supplementary Figure 14. Phylogenetic tree of N-acetylmuramoyl-L-alanine amidase.** The ML tree was inferred using 167 amino acids derived from 105 OTUs with a WAG+F+I+G4 substitution model. Bootstrap supports (BP) are shown on each node.

**Supplementary Table 1.** List of the *Braarudosphaera bigelowii* strains used in this study.

| Sampling site | latitude/longitude | Sampling day | Purpose | Strains | Note |
| --- | --- | --- | --- | --- | --- |
| Asamushi, Aomori, Japan | 40°53'37.2"N 140°51'32.5"E | 1990/6/18 | establishing strains, observation | MK90-06 (extinct now) | Cells possessed an endosymbiont. |
| Tomari Port, Tottori, Japan | 35°31'01.7"N 133°56'14.8"E | 2014/6/6 | Sequencing the endosymbiont genome | Not established | Cells possessed an endosymbiont. |
| Ikenoura Port, Kochi, Japan | 33°24'34.9"N 133°24'45.0"E | 2015/5/17 | establishing strains, observation, RNA-seq | KC1-P2 (NIES-3865) | Cells possessed an endosymbiont but lost it in the beginning of culture experiments. |
|  |  | 2017/5/24 | establishing strains, observation | KC15-24 (NIES-4442) | Cells did not possess an endosymbiont. |

**Supplementary Table 2.** List of the selected substitution models for cyanobacterial phylogenetic analysis.

| partition name | position | substitution model |
| --- | --- | --- |
| P1 | 1-147 | LG+I+G4 |
| P2 | 148-309 | LG+G4 |
| P3 | 310-573 | LG+I+G4 |
| P4 | 574-1002 | LG+I+G4 |
| P5 | 1003-1606 | LG+I+G4 |
| P6 | 1607-2193 | LG+I+G4 |
| P7 | 2194-2420 | LG+I+G4 |
| P8 | 2421-2676 | LG+I+G4 |
| P9 | 2677-2826 | LG+I+G4 |
| P10 | 2827-3153 | LG+I+G4 |
| P11 | 3154-3407 | LG+I+G4 |
| P12 | 3408-3662 | LG+I+G4 |
| P13 | 3663-4054 | LG+I+G4 |
| P14 | 4055-4326 | LG+I+G4 |
| P15 | 4327-4605 | LG+I+G4 |
| P16 | 4606-4699 | MTZOA+G4 |
| P17 | 4700-5112 | LG+I+G4 |
| P18 | 5113-5269 | LG+G4 |
| P19 | 5270-5479 | LG+I+G4 |
| P20 | 5480-5570 | LG+G4 |
| P21 | 5571-5698 | LG+G4 |
| P22 | 5699-6005 | LG+I+G4 |
| P23 | 6006-6136 | LG+I+G4 |
| P24 | 6137-6214 | WAG+G4 |
| P25 | 6215-6746 | LG+I+G4 |
| P26 | 6747-6820 | MTZOA+G4 |
| P27 | 6821-7416 | LG+I+G4 |
| P28 | 7417-7813 | LG+I+G4 |
| P29 | 7814-8239 | LG+I+G4 |
| P30 | 8240-8617 | LG+I+G4 |
| P31 | 8618-8740 | LG+I+G4 |
| P32 | 8741-8981 | LG+I+G4 |
| P33 | 8982-9085 | LG+I+G4 |
| P34 | 9086-9620 | LG+I+G4 |
| P35 | 9621-9774 | LG+G4 |
| P36 | 9775-10267 | LG+I+G4 |
| P37 | 10268-10592 | LG+I+G4 |
| P38 | 10593-10753 | LG+G4 |
| P39 | 10754-10944 | LG+G4 |
| P40 | 10945-11234 | MTZOA+I+G4+F |
| P41 | 11235-11327 | JTT-DCMUT+I+G4 |
| P42 | 11328-11715 | LG+I+G4 |
| P43 | 11716-11941 | LG+I+G4 |
| P44 | 11942-12329 | LG+I+G4 |
| P45 | 12330-12540 | LG+G4 |
| P46 | 12541-12783 | LG+I+G4 |
| P47 | 12784-13105 | LG+I+G4 |
| P48 | 13106-13288 | LG+I+G4 |
| P49 | 13289-13542 | LG+G4+F |
| P50 | 13543-14000 | LG+I+G4+F |
| P51 | 14001-14405 | LG+G4 |
| P52 | 14406-14650 | LG+I+G4 |
| P53 | 14651-14743 | LG+G4 |
| P54 | 14744-15091 | MTZOA+I+G4+F |
| P55 | 15092-15227 | MTZOA+G4+F |
| P56 | 15228-15804 | LG+I+G4 |
| P57 | 15805-15983 | LG+I+G4 |
| P58 | 15984-16116 | CPREV+I+G4 |
| P59 | 16117-16540 | LG+I+G4 |
| P60 | 16541-16988 | LG+I+G4 |
| P61 | 16989-17334 | LG+I+G4 |
| P62 | 17335-17622 | LG+I+G4+F |
| P63 | 17623-17836 | LG+G4 |
| P64 | 17837-18042 | LG+I+G4 |
| P65 | 18043-18162 | CPREV+I+G4 |
| P66 | 18163-18567 | LG+I+G4 |
| P67 | 18568-18706 | LG+G4 |
| P68 | 18707-19175 | LG+I+G4 |
| P69 | 19176-19747 | LG+I+G4 |
| P70 | 19748-20065 | LG+I+G4 |
| P71 | 20066-20297 | LG+I+G4 |
| P72 | 20298-20705 | LG+I+G4 |
| P73 | 20706-20804 | LG+I+G4 |
| P74 | 20805-21720 | LG+I+G4 |
| P75 | 21721-22106 | LG+I+G4 |
| P76 | 22107-22608 | LG+I+G4+F |
| P77 | 22609-22737 | LG+G4 |
| P78 | 22738-22851 | LG+I+G4 |
| P79 | 22852-23036 | LG+I+G4 |
| P80 | 23037-23283 | LG+I+G4 |
| P81 | 23284-23390 | LG+G4 |
| P82 | 23391-23794 | LG+G4 |
| P83 | 23795-24251 | LG+I+G4 |
| P84 | 24252-24561 | LG+I+G4 |
| P85 | 24562-24715 | MTZOA+G4 |
| P86 | 24716-25072 | LG+I+G4 |
| P87 | 25073-25157 | LG+I+G4 |
| P88 | 25158-25482 | LG+I+G4 |
| P89 | 25483-25646 | LG+G4 |
| P90 | 25647-25851 | JTT+I+G4 |
| P91 | 25852-25918 | RTREV+G4 |
| P92 | 25919-26152 | LG+I+G4 |
| P93 | 26153-26655 | LG+I+G4 |
| P94 | 26656-26820 | LG+I+G4 |
| P95 | 26821-26963 | LG+I+G4 |
| P96 | 26964-27093 | LG+G4 |
| P97 | 27094-27340 | LG+I+G4 |
| P98 | 27341-27446 | LG+I+G4 |
| P99 | 27447-27831 | LG+I+G4 |
| P100 | 27832-27955 | LG+G4 |
| P101 | 27956-28433 | LG+I+G4 |
| P102 | 28434-28771 | LG+I+G4 |
| P103 | 28772-28899 | LG+I+G4 |
| P104 | 28900-29992 | LG+I+G4 |
| P105 | 29993-30216 | LG+I+G4 |
| P106 | 30217-30557 | LG+I+G4 |
| P107 | 30558-30763 | LG+I+G4+F |
| P108 | 30764-31214 | LG+I+G4 |
| P109 | 31215-32015 | LG+I+G4 |
| P110 | 32016-32306 | LG+I+G4 |
| P111 | 32307-32703 | LG+I+G4 |
| P112 | 32704-33038 | LG+I+G4 |
| P113 | 33039-33482 | LG+I+G4 |
| P114 | 33483-33764 | LG+I+G4 |
| P115 | 33765-33828 | MTZOA+G4 |
| P116 | 33829-34001 | LG+I+G4 |
| P117 | 34002-34379 | LG+I+G4 |
| P118 | 34380-34666 | LG+I+G4 |
| P119 | 34667-34887 | JTT+I+G4 |
| P120 | 34888-35141 | LG+I+G4 |
| P121 | 35142-35467 | LG+G4 |
| P122 | 35468-35616 | LG+I+G4 |
| P123 | 35617-35983 | LG+I+G4 |
| P124 | 35984-36142 | MTZOA+I+G4 |
| P125 | 36143-36257 | LG+I+G4 |
| P126 | 36258-36753 | LG+G4 |
| P127 | 36754-36829 | LG+G4 |
| P128 | 36830-37016 | LG+I+G4 |
| P129 | 37017-37351 | LG+I+G4 |
| P130 | 37352-37645 | LG+I+G4 |
| P131 | 37646-38209 | LG+I+G4 |
| P132 | 38210-38334 | LG+I+G4 |
| P133 | 38335-38656 | LG+G4 |
| P134 | 38657-39082 | LG+I+G4 |
| P135 | 39083-39446 | LG+I+G4 |
| P136 | 39447-39688 | LG+I+G4 |
| P137 | 39689-40222 | LG+I+G4 |
| P138 | 40223-40364 | LG+I+G4 |
| P139 | 40365-40732 | LG+I+G4 |
| P140 | 40733-40970 | MTZOA+I+G4+F |
| P141 | 40971-41295 | LG+I+G4 |
| P142 | 41296-41428 | LG+G4 |
| P143 | 41429-41523 | LG+G4 |
| P144 | 41524-41611 | LG+G4 |
| P145 | 41612-42019 | LG+I+G4 |
| P146 | 42020-42269 | LG+I+G4+F |
| P147 | 42270-42471 | LG+G4 |
| P148 | 42472-43059 | LG+I+G4 |
| P149 | 43060-43132 | LG+I+G4 |
| P150 | 43133-43360 | LG+I+G4 |
| P151 | 43361-43668 | LG+I+G4 |
| P152 | 43669-44019 | LG+I+G4 |
| P153 | 44020-44292 | LG+I+G4 |
| P154 | 44293-44381 | LG+G4 |
| P155 | 44382-45044 | LG+I+G4 |
| P156 | 45045-45881 | LG+I+G4 |
| P157 | 45882-46002 | LG+G4 |
| P158 | 46003-46240 | LG+I+G4 |
| P159 | 46241-46297 | LG+G4 |
| P160 | 46298-46587 | LG+I+G4 |

**Supplementary Table 3.** List of samples for RNA-seq

| **sample ID** | **sequencing length** | **sequencing size** | **medium** | **light condition** |
| --- | --- | --- | --- | --- |
| KC1-P2-N | 152133258 | 18060856420 | ESM-N | light/dark phase (mixed) |
| KC1-P2_N1 | 18657344 | 2220319374 | ESM | light phase |
| KC1-P2_N2 | 17587804 | 2098578224 | ESM | dark phase |
| KC1-P2_N3 | 16751734 | 2000971101 | ESM-N | light phase |
| KC1-P2_N4 | 14706592 | 1756744483 | ESM-N | dark phase |

**Supplementary Table 4.** Statistics related to the genomes of the *Braarudosphaera bigelowii* endosymbiont and UCYN-A.

|  | *B. bigelowii* endoymbiont | UCYN-A1* | UCYN-A2* |
| --- | --- | --- | --- |
| genome size | 1491611 | 1443806 | 1485499 |
| Number of contigs | 1 | 1 | 52 |
| GC% | 33 | 31 | 31 |
| Number of CDSs | 1188** | 1200 | 1246 |
| Number of tRNAs | 37 | 36 | 37 |
| Number of rRNAs | 6 | 6 | 6 |
| *based on Bomber et al. (2014). | |  |  |
| **excluding putative pseudogenes. | |  |  |

**Supplementary Table 5.** List of unique genes of the *Braarudosphaera bigelowii* endosymbiont.

| Gene ID | Annotation | | Note | |
| --- | --- | --- | --- | --- |
| Unique genes lacked in UCYN-A1 | | | | |
| CPSB_00047 | | hypothetical protein | |  |
| CPSB_00051 | | hypothetical protein | |  |
| CPSB_00077 | | 30S ribosomal protein S17 | | pseudogene in UCYN-A1 |
| CPSB_00098 | | Aminomethyltransferase | |  |
| CPSB_00160 | | hypothetical protein | | the same position and size as UCYN_11200 of UCYN-A1 |
| CPSB_00167 | | Beta-lactamase hydrolase-like protein | |  |
| CPSB_00190 | | hypothetical protein | | pseudogene in UCYN-A1 |
| CPSB_00192 | | 7-cyano-7-deazaguanine synthase | | pseudogene in UCYN-A1 |
| CPSB_00203 | | Thymidylate kinase | |  |
| CPSB_00270 | | hypothetical protein | |  |
| CPSB_00284 | | hypothetical protein | |  |
| CPSB_00290 | | 2-succinylbenzoate--CoA ligase | | pseudogene in UCYN-A1 |
| CPSB_1_0011 | | hypothetical protein | |  |
| CPSB_00356 | | hypothetical protein | |  |
| CPSB_00361 | | hypothetical protein | | pseudogene in UCYN-A1 |
| CPSB_00362 | | Bifunctional ligase/repressor BirA | | pseudogene in UCYN-A1 |
| CPSB_00363 | | hypothetical protein | |  |
| CPSB_00392 | | hypothetical protein | |  |
| CPSB_00449 | | hypothetical protein | |  |
| CPSB_00497 | | hypothetical protein | |  |
| CPSB_00609 | | hypothetical protein | |  |
| CPSB_00626 | | hypothetical protein | |  |
| CPSB_00757 | | hypothetical protein | |  |
| CPSB_00816 | | Glucosylglycerol-phosphate phosphatase | |  |
| CPSB_00861 | | Biosynthetic arginine decarboxylase | | pseudogene in UCYN-A1 |
| CPSB_00895 | | hypothetical protein | |  |
| CPSB_00900 | | Modification methylase DpnIIA | | pseudogene in UCYN-A1 |
| CPSB_00908 | | hypothetical protein | |  |
| CPSB_00927 | | Lon protease 2 | | pseudogene in UCYN-A1 |
| CPSB_00930 | | hypothetical protein | |  |
| CPSB_00931 | | hypothetical protein | |  |
| CPSB_00945 | | Nicotinamide-nucleotide amidohydrolase PncC | | pseudogene in UCYN-A1 |
| CPSB_00985 | | Folate-biopterin transporter | |  |
| CPSB_01016 | | hypothetical protein | |  |
| CPSB_01029 | | Threonylcarbamoyl-AMP synthase | | pseudogene in UCYN-A1 |
| CPSB_01041 | | hypothetical protein | |  |
| CPSB_01118 | | tetratricopeptide repeat protein | |  |
| CPSB_01171 | | hypothetical protein | |  |
| CPSB_01198 | | hypothetical protein | | pseudogene in UCYN-A1 |
| CPSB_01243 | | hypothetical protein | |  |
| Unique genes lacked in UCYN-A2 | | | | |
| CPSB_00179 | | lysS, Lysine--tRNA ligase | | pseudogene (ucyna2_01246+ucyna2_01246) of UCYN-A2 |
| CPSB_00539 | | hypothetical protein | | pseudogene(ucyna2_00733+ucyna2_00734) of UCYN-A2 |
| CPSB_00812 | | carB, Carbamoyl-phosphate synthase large chain | | pseudogene(ucyna2_00191+ucyna2_00192) of UCYN-A2 |
| CPSB_00905 | | hypothetical protein | | putative inter-contigs region |
| CPSB_00988 | | hypothetical protein | | pseudogene(ucyna2_01126-01122) of UCYN-A2 |

**Supplementary Table 6.** List of unique genes of UCYN-A1.

| Gene ID | Annotation | Note |
| --- | --- | --- |
| UCYN_00310 | restriction endonuclease |  |
| UCYN_00430 | DNA replication and repair protein RecO | pseudogene in the *B. bigelowii* endosymbiont |
| UCYN_01700 | hypothetical protein |  |
| UCYN_01800 | hypothetical protein |  |
| UCYN_02600 | hypothetical protein |  |
| UCYN_02740 | hypothetical protein |  |
| UCYN_02800 | hypothetical protein |  |
| UCYN_03740 | hypothetical protein |  |
| UCYN_03890 | glycerol dehydrogenase-like oxidoreductase | pseudogene in the *B. bigelowii* endosymbiont |
| UCYN_04150 | hypothetical protein |  |
| UCYN_04170 | hypothetical protein |  |
| UCYN_04260 | hypothetical protein |  |
| UCYN_04440 | hypothetical protein |  |
| UCYN_04700 | HAS barrel domain protein | pseudogene in the *B. bigelowii* endosymbiont |
| UCYN_04840 | phosphopantetheinyl transferase | pseudogene in the *B. bigelowii* endosymbiont |
| UCYN_05340 | hypothetical protein |  |
| UCYN_05770 | NurA domain-containing protein |  |
| UCYN_06100 | hypothetical protein |  |
| UCYN_07180 | hypothetical protein |  |
| UCYN_07550 | hypothetical protein |  |
| UCYN_07560 | hypothetical protein |  |
| UCYN_08910 | chlorophyll a/b binding light-harvesting protein | pseudogene in the *B. bigelowii* endosymbiont |
| UCYN_09090 | hypothetical protein |  |
| UCYN_09380 | transcriptional regulator%2C GntR family | pseudogene in the *B. bigelowii* endosymbiont |
| UCYN_09460 | hypothetical protein |  |
| UCYN_09550 | site-specific recombinase XerD |  |
| UCYN_09560 | predicted transcriptional regulator |  |
| UCYN_09970 | hypothetical protein |  |
| UCYN_10260 | hypothetical protein |  |
| UCYN_10790 | hypothetical protein |  |
| UCYN_10920 | NAD-dependent aldehyde dehydrogenase |  |
| UCYN_11200 | hypothetical protein | the same position and size as CPSB_00160 of the *B. bigelowii* endosymbiont |
| UCYN_11540 | Ycf66 protein N-terminus | pseudogene in the *B. bigelowii* endosymbiont |
| UCYN_11840 | uncharacterized conserved protein | pseudogene in the *B. bigelowii* endosymbiont |
| UCYN_11920 | hypothetical protein |  |
| UCYN_12100 | predicted ATPase | pseudogene in the *B. bigelowii* endosymbiont |

**Supplementary Table 7.** List of rRNA contigs in RNA-seq.

| **Contig ID** | **blast tophit (excl. environmental sequence)*** | **Identity (%)** |
| --- | --- | --- |
| KC1-P2-N_CL7801Contig1_1 | *Braaudosphaera bigeloweii* 18S rRNA | 100 |
| KC1-P2-N_k25_Locus_5431_Transcript_7_1** | *Mesorhizobium* sp. strain 2_9_6_1 16S rRNA | 100 |
| KC1-P2_N1_k31_Locus_8572_Transcript_1_1 | *Braaudosphaera bigeloweii* platid 16S rRNA | 99.8 |
| KC1-P2_N1_k43_Locus_8416_Transcript_1_1 | *Braaudosphaera bigeloweii platid 16S rRNA* | 99.8 |
| KC1-P2_N3_CL1545Contig1_1 | *Braaudosphaera bigeloweii* 18S rRNA | 100 |
| KC1-P2_N3_CL4100Contig1_1 | Actinobacterium SM45 16S rRNA | 99.6 |
| KC1-P2_N3_CL4938Contig1_1 | *Kytococcus sedentarius* | 99.1 |
| KC1-P2_N3_CL5225Contig1_1 | *Braaudosphaera bigeloweii* platid 16S rRNA | 99.3 |
| *Blastn to the NCBI refseq database. |  |  |
| ** removed chimera regions. |  |  |

**Supplementary Table 8.** List of genes for nitrogen metabolism.

| **Function** | **Gene name** | **Annotation** | **EC number** | **Gene/transcript ID** | | | **Presence of RNA-seq reads?**** |
| --- | --- | --- | --- | --- | --- | --- | --- |
|  |  |  |  | ***B. bigelowii*** | ***E. huxleyi*** | ***C. tobinii*** |  |
| Nitrate/Nitrite uptake and metabolism |  |  |  |  |  |  |  |
|  | Nrt | MFS transporter | - |  | EOD23024 | KOO21256.1 | No |
|  |  |  |  |  | EOD28141 |  | No |
|  |  |  |  |  | EOD10459 |  | No |
|  |  |  |  |  | EOD39142 |  | No |
|  |  |  |  |  | EOD19722? |  | No |
|  |  |  |  |  | EOD39011 |  | No |
|  |  |  |  |  | EOD36145 |  | No |
|  |  |  |  |  | EOD20301 |  | Yes, but not homolog |
|  |  |  |  |  | EOD15007 |  | No |
|  |  |  |  |  | EOD26192 |  | Yes, but not homolog |
|  |  |  |  |  | EOD26193 |  | No |
|  |  |  |  |  | EOD38169 |  | No |
|  | NR | nitrate reductase | EC:1.7.1.1; 1.7.1.2; 1.7.1.3 |  | * | KOO21257.1 |  |
|  | NAR1 | formate/nitrite transporter |  | KC1-P2-N_k31_Locus_10954_Transcript_1_1 | EOD26494 | KOO21263.1 | No |
|  |  |  |  | KC1-P2-N_k43_Locus_22399_Transcript_2_1 | EOD04037 | KOO21572.1 | No |
|  |  |  |  |  | EOD30801 | KOO25737.1 | No |
|  |  |  |  |  | EOD21933 | KOO32765.1 | No |
|  |  |  |  |  | EOD32330 | KOO33535.1 | No |
|  |  |  |  |  | EOD30192 | KOO35922.1 | No |
|  |  |  |  |  |  |  |  |
|  |  |  |  |  |  |  |  |
|  | NirA | ferredoxin-nitrite reductase | EC:1.7.7.1 |  | EOD24873 | KOO21258.1 | Yes, but not homolog |
|  |  |  |  |  | EOD38616 | KOO21959.1 | Yes, but not homolog |
|  |  |  |  |  |  |  |  |
|  |  |  |  |  |  |  |  |
| Nitroalkane uptake | ncd2 | nitronate monooxygenase | EC:1.13.12.16 | KC1-P2-N_k25_Locus_16932_Transcript_1_1 | EOD05723 |  | No |
|  |  |  |  |  | EOD40342 |  | Yes |
|  |  |  |  |  | EOD10762 |  | Yes |
|  |  |  |  |  |  |  |  |
| Formamide reduction |  | formamidase | EC:3.5.1.49 |  | EOD05116 | KOO32084.1 | No |
|  |  |  |  |  | EOD20197 |  | No |
|  |  |  |  |  | EOD15482 |  | No |
|  |  |  |  |  | EOD10498 |  | No |
|  |  |  |  |  | EOD35659 |  | No |
|  |  |  |  |  | EOD38790 |  | No |
|  |  |  |  |  | EOD20755 |  | No |
|  |  |  |  |  | EOD20753 |  | No |
|  |  |  |  |  |  |  |  |
| Glutamate metabolism | glnA | glutamine synthetase | EC:6.3.1.2 | KC1-P2-N_CL2862Contig1_1 | EOD13159 | KOO21867.1 | Yes |
|  |  |  |  | KC1-P2-N_CL8516Contig1_1 | EOD36680 | KOO23941.1 | Yes |
|  |  |  |  | KC1-P2-N_k49_Locus_8643_Transcript_1_1 | EOD04060 | KOO30748.1 | Yes |
|  |  |  |  | KC1-P2-N_k55_Locus_10369_Transcript_2_1 | EOD37314 | KOO53340.1 | No |
|  |  |  |  | KC1-P2-N_k55_Locus_20141_Transcript_3_1 | EOD19857 |  | Yes |
|  |  |  |  | KC1-P2_N1_k25_Locus_16717_Transcript_1_1 | EOD28928 |  | Yes |
|  |  |  |  | KC1-P2_N1_k31_Locus_13154_Transcript_1_1 | EOD19170 |  | Yes |
|  |  |  |  | KC1-P2_N2_CL1345Contig1_1 |  |  |  |
|  |  |  |  | KC1-P2_N2_k25_Locus_73_Transcript_1_1 |  |  |  |
|  |  |  |  | KC1-P2_N3_k25_Locus_4906_Transcript_1_1 |  |  |  |
|  |  |  |  | KC1-P2_N3_k31_Locus_2473_Transcript_2_1 |  |  |  |
|  |  |  |  | KC1-P2_N4_k49_Locus_4149_Transcript_1_1 |  |  |  |
|  | gltD | glutamate synthase (NADPH) small chain | EC:1.4.1.13 | KC1-P2-N_k31_Locus_14987_Transcript_1_1 | EOD33253 |  | Yes |
|  |  |  |  |  | EOD23577 |  | Yes |
|  | GLT1 | glutamate synthase (NADH) | EC:1.4.1.14 | KC1-P2-N_k25_Locus_9482_Transcript_1_1 |  |  |  |
|  | GLU | glutamate synthase (ferredoxin) | EC:1.4.7.1 | KC1-P2-N_k25_Locus_9482_Transcript_1_1 | EOD37258 | KOO22112.1 | Yes |
|  |  |  |  | KC1-P2_N2_k37_Locus_2688_Transcript_6_1 | EOD35180 |  | Yes |
|  | GDH2 | glutamate dehydrogenase | EC:1.4.1.2 |  | EOD36944 | KOO34829.1 | No |
|  | glsA | glutaminase | EC:3.5.1.2 |  | EOD11679 |  | Yes, but not homolog |
|  |  |  |  |  | EOD24705 |  | No |
|  |  |  |  |  | EOD08324 |  | No |
|  |  |  |  |  | EOD27671 |  | Yes, but not homolog |
|  |  |  |  |  |  |  |  |
| Cyanate uptake | cynS | cyanate lyase | EC:4.2.1.104 |  | EOD40527 |  | No |
|  |  |  |  |  | EOD25034 |  | No |
|  |  |  |  |  |  |  |  |
| Arginine metabolism | CPS1 | carbamoyl-phosphate synthase (ammonia) | EC:6.3.4.16 | KC1-P2-N_k25_Locus_1757_Transcript_15_1 | EOD13769 | KOO21315.1 | Yes |
|  |  |  |  | KC1-P2-N_k31_Locus_3147_Transcript_16_1 |  |  |  |
|  |  |  |  | KC1-P2_N1_k43_Locus_3475_Transcript_1_1 |  |  |  |
|  | GOT1 | aspartate aminotransferase, cytoplasmic | EC:2.6.1.1 |  | EOD22514? |  | Yes, but not homolog |
|  |  |  |  |  | EOD11943 |  | Yes, but not homolog |
|  | GOT2 | aspartate aminotransferase, mitochondrial | EC:2.6.1.1 | KC1-P2_N1_k37_Locus_13362_Transcript_1_1 | EOD32089 | KOO20853.1 | Yes |
|  |  |  |  | KC1-P2_N3_k31_Locus_11758_Transcript_6_1 | EOD23990 | KOO21330.1 | Yes |
|  |  |  |  |  |  | KOO32397.1 |  |
|  |  | acetylornithine transaminase | EC:2.6.1.11 | KC1-P2_N1_k43_Locus_2087_Transcript_1_1 |  |  |  |
|  |  |  |  | KC1-P2-N_CL1160Contig1_1 |  |  |  |
|  | argE | acetylornithine deacetylase | EC:3.5.1.16 | KC1-P2-N_k49_Locus_22685_Transcript_3_1 |  |  |  |
|  |  |  |  | KC1-P2-N_k65_Locus_23870_Transcript_1_1 |  |  |  |
|  | argJ | glutamate N-acetyltransferase / amino-acid N-acetyltransferase | EC:2.3.1.35; 2.3.1.1 | KC1-P2-N_k25_Locus_4230_Transcript_8_1 | EOD31388 | KOO22589.1 | Yes |
|  |  |  |  | KC1-P2-N_k49_Locus_17801_Transcript_3_1 |  |  |  |
|  |  |  |  | KC1-P2-N_k61_Locus_16935_Transcript_1_1 |  |  |  |
|  | OTC | ornithine carbamoyltransferase | EC:2.1.3.3 | KC1-P2-N_k37_Locus_9158_Transcript_3_1 | EOD28327 |  | Yes |
|  |  |  |  |  | EOD26789 |  | Yes |
|  | argG | argininosuccinate synthase | EC:6.3.4.5 |  | EOD34470 | KOO31298.1 | No |
|  |  |  |  |  | EOD09871 |  | No |
|  | argH | argininosuccinate lyase | EC:4.3.2.1 | KC1-P2-N_k49_Locus_12751_Transcript_2_1 | EOD30692 | KOO27328.1 | Yes |
|  |  |  |  |  | EOD08960 |  | Yes |
|  |  |  |  |  | EOD08961 |  | Yes |
|  | NOA1 | nitric-oxide synthase, plant | EC:1.14.13.39 | KC1-P2-N_k49_Locus_1949_Transcript_1_1 | EOD14979 | KOO31498.1 | Yes |
|  | arg | arginase | EC:3.5.3.1 |  | EOD22446 |  | Yes, but not homolog |
|  |  |  |  |  | EOD28607 |  | No |
|  |  |  |  |  | EOD23607 |  | No |
|  |  |  |  |  | EOD17790 |  | Yes, but not homolog |
|  | URE | urease | EC:3.5.1.5 |  | EOD32519 | KOO24765.1 | Yes, but contaminated bacterial homolog |
|  |  |  |  |  | EOD34013 |  | Yes, but contaminated bacterial homolog |
|  |  |  |  |  |  |  |  |
| Urea uptake | URT | urea transporter | - |  | EOD41148 | KOO22511.1 | No |
|  |  |  |  |  |  |  |  |
| Ammonia uptake | DUR | ammonia transporter | - | KC1-P2-N_CL3922Contig1_1 | EOD11231 | KOO30162.1 | No |
|  |  |  |  | KC1-P2-N_k25_Locus_16869_Transcript_1_1 | EOD14540 | KOO29747.1 | No |
|  |  |  |  | KC1-P2-N_k25_Locus_3802_Transcript_72_1 | EOD26435 |  | No |
|  |  |  |  | KC1-P2-N_k25_Locus_7273_Transcript_10_1 | EOD36061 |  | No |
|  |  |  |  | KC1-P2-N_k31_Locus_8488_Transcript_3_1 | EOD30791 |  | No |
|  |  |  |  | KC1-P2-N_k43_Locus_11606_Transcript_3_1 | EOD36369 |  | No |
|  |  |  |  | KC1-P2-N_k43_Locus_18117_Transcript_1_1 | EOD30373 |  | No |
|  |  |  |  | KC1-P2-N_k49_Locus_13283_Transcript_4_1 | EOD08673 |  | No |
|  |  |  |  | KC1-P2-N_k61_Locus_9395_Transcript_6_1 | EOD17395 |  | No |
|  |  |  |  | KC1-P2-N_k69_Locus_8612_Transcript_3_1 | EOD31368 |  | No |
|  |  |  |  | KC1-P2_N1_CL4324Contig1_1 | EOD37853 |  | No |
|  |  |  |  | KC1-P2_N1_k49_Locus_7401_Transcript_1_1 | EOD38381 |  | No |
|  |  |  |  | KC1-P2_N1_k55_Locus_365_Transcript_1_1 | EOD25529 |  | No |
|  |  |  |  | KC1-P2_N2_k25_Locus_10816_Transcript_3_1 | EOD35388 |  | No |
|  |  |  |  | KC1-P2_N3_k49_Locus_8953_Transcript_1_1 | EOD31145 |  | No |
|  |  |  |  | KC1-P2_N4_k37_Locus_3169_Transcript_1_1 | EOD41738 |  | No |
|  |  |  |  |  | EOD41737 |  | No |
|  |  |  |  |  | EOD37849 |  | No |
|  |  |  |  |  | EOD09539 |  | No |
|  |  |  |  |  | EOD27895 |  | No |
| *We could not find NR genes in the *E. huxlei* genome, but NR activity of *E. huxleyi* is shown by Iwamoto and Shiraiwa (2003). | | | | | | | |
| **We performed tblastn search of the genes of *E. huxleyi* against the non-redundant raw reads of *B. bigelowii* RNA-seq with a cut-off: e-value <1e-3. | | | | | | | |

**Supplementary Table 9**. Relative expression values of nitrogen metabolism-related genes in *Braarudosphaera bigelowii*.

| **Gene name** | **Annotation** | **Gene/transcript ID** | **Relative expression values (RPKM)** | | | | **Ranks of RPKM in total transcripts*** | | | |
| --- | --- | --- | --- | --- | --- | --- | --- | --- | --- | --- |
|  |  | ***B. bigelowii*** | **N+**  **light** | **N+**  **dark** | **N-light** | **N-dark** | **N+light** | **N+dark** | **N-light** | **N-dark** |
| NAR1 | formate/nitrite transporter | KC1-P2-N_k31_Locus_10954_Transcript_1_1 | 4.6 | 4.9 | 27.2 | 26.2 | 18890 | 17848 | 3677 | 3891 |
|  |  | KC1-P2-N_k43_Locus_22399_Transcript_2_1 | 4.4 | 2.2 | 6.8 | 7.2 | 19457 | 29977 | 15320 | 14539 |
| ncd2 | nitronate monooxygenase | KC1-P2-N_k25_Locus_16932_Transcript_1_1 | 0.9 | 0.6 | 1.4 | 1.5 | 41240 | 43470 | 42119 | 41048 |
| glnA | glutamine synthetase | KC1-P2-N_CL2862Contig1_1 | 2.8 | 3.0 | 14.0 | 12.7 | 26221 | 25011 | 7790 | 8649 |
|  |  | KC1-P2-N_CL8516Contig1_1 | 53.0 | 49.9 | 56.2 | 55.7 | 1781 | 1936 | 1458 | 1494 |
|  |  | KC1-P2-N_k49_Locus_8643_Transcript_1_1 | 596.4 | 617.4 | 2.7 | 2.5 | 46 | 44 | 31765 | 33091 |
|  |  | KC1-P2-N_k55_Locus_10369_Transcript_2_1 | 4.7 | 5.5 | 17.2 | 17.2 | 18627 | 16279 | 6207 | 6249 |
|  |  | KC1-P2-N_k55_Locus_20141_Transcript_3_1 | 0.1 | 0.3 | 5.0 | 5.5 | 48784 | 46767 | 19728 | 18331 |
|  |  | KC1-P2_N1_k25_Locus_16717_Transcript_1_1 | 6.0 | 7.3 | 48.0 | 55.5 | 15471 | 12965 | 1808 | 1499 |
|  |  | KC1-P2_N1_k31_Locus_13154_Transcript_1_1 | 10.5 | 8.5 | 31.5 | 30.5 | 9708 | 11449 | 3082 | 3218 |
|  |  | KC1-P2_N2_CL1345Contig1_1 | 4.3 | 4.2 | 17.0 | 15.9 | 19915 | 19913 | 6287 | 6790 |
|  |  | KC1-P2_N2_k25_Locus_73_Transcript_1_1 | 36.8 | 45.0 | 29.1 | 32.0 | 2654 | 2161 | 3402 | 3023 |
|  |  | KC1-P2_N3_k25_Locus_4906_Transcript_1_1 | 0.8 | 0.7 | 7.4 | 6.2 | 42722 | 42279 | 14329 | 16478 |
|  |  | KC1-P2_N3_k31_Locus_2473_Transcript_2_1 | 2.7 | 3.6 | 19.6 | 16.7 | 26892 | 22207 | 5357 | 6476 |
|  |  | KC1-P2_N4_k49_Locus_4149_Transcript_1_1 | 5.3 | 5.2 | 32.7 | 30.6 | 17071 | 17055 | 2939 | 3196 |
| gltD | glutamate synthase (NADPH) small chain | KC1-P2-N_k31_Locus_14987_Transcript_1_1 | 7.1 | 13.3 | 11.7 | 11.5 | 13562 | 7653 | 9304 | 9508 |
| GLT1 | glutamate synthase (NADH) | KC1-P2-N_k25_Locus_9482_Transcript_1_1 | 23.8 | 34.3 | 15.2 | 12.5 | 4329 | 2909 | 7113 | 8757 |
| GLU | glutamate synthase (ferredoxin) | KC1-P2-N_k25_Locus_9482_Transcript_1_1 | 23.8 | 34.3 | 15.2 | 12.5 | 4329 | 2909 | 7113 | 8757 |
|  |  | KC1-P2_N2_k37_Locus_2688_Transcript_6_1 | 199.8 | 227.6 | 0.6 | 1.2 | 307 | 270 | 47700 | 43581 |
| CPS1 | carbamoyl-phosphate synthase (ammonia) | KC1-P2-N_k25_Locus_1757_Transcript_15_1 | 13.2 | 15.8 | 19.0 | 19.1 | 7811 | 6441 | 5561 | 5563 |
|  |  | KC1-P2-N_k31_Locus_3147_Transcript_16_1 | 19.8 | 25.8 | 9.4 | 9.4 | 5211 | 3975 | 11582 | 11496 |
|  |  | KC1-P2_N1_k43_Locus_3475_Transcript_1_1 | 38.9 | 47.4 | 4.7 | 5.1 | 2503 | 2043 | 20960 | 19437 |
| GOT2 | aspartate aminotransferase, mitochondrial | KC1-P2_N1_k37_Locus_13362_Transcript_1_1 | 17.3 | 24.1 | 4.6 | 4.4 | 6005 | 4279 | 21420 | 21992 |
|  |  | KC1-P2_N3_k31_Locus_11758_Transcript_6_1 | 21.4 | 11.0 | 34.6 | 37.8 | 4831 | 9067 | 2774 | 2492 |
|  | acetylornithine transaminase | KC1-P2_N1_k43_Locus_2087_Transcript_1_1 | 31.3 | 31.2 | 12.4 | 15.0 | 3191 | 3251 | 8836 | 7236 |
|  |  | KC1-P2-N_CL1160Contig1_1 | 2.3 | 1.4 | 1.2 | 1.7 | 29725 | 36689 | 43855 | 39131 |
| argE | acetylornithine deacetylase | KC1-P2-N_k49_Locus_22685_Transcript_3_1 | 5.3 | 5.5 | 9.8 | 9.9 | 17041 | 16363 | 11023 | 10907 |
|  |  | KC1-P2-N_k65_Locus_23870_Transcript_1_1 | 0.1 | 0.0 | 13.7 | 15.9 | 49429 | 50439 | 7926 | 6794 |
| argJ | glutamate N-acetyltransferase / amino-acid N-acetyltransferase | KC1-P2-N_k25_Locus_4230_Transcript_8_1 | 5.5 | 4.5 | 6.6 | 7.5 | 16495 | 19153 | 15665 | 13937 |
|  |  | KC1-P2-N_k49_Locus_17801_Transcript_3_1 | 3.4 | 3.6 | 5.3 | 5.1 | 23098 | 22121 | 18911 | 19535 |
|  |  | KC1-P2-N_k61_Locus_16935_Transcript_1_1 | 7.7 | 5.6 | 10.7 | 10.8 | 12605 | 16044 | 10132 | 10070 |
| OTC | ornithine carbamoyltransferase | KC1-P2-N_k37_Locus_9158_Transcript_3_1 | 30.9 | 30.3 | 8.8 | 7.4 | 3246 | 3357 | 12269 | 14171 |
| argH | argininosuccinate lyase | KC1-P2-N_k49_Locus_12751_Transcript_2_1 | 66.8 | 71.9 | 0.2 | 0.4 | 1372 | 1293 | 49767 | 48902 |
| DUR | ammonia transporter | KC1-P2-N_CL3922Contig1_1 | 58.5 | 83.2 | 67.7 | 56.9 | 1580 | 1092 | 1149 | 1461 |
|  |  | KC1-P2-N_k25_Locus_16869_Transcript_1_1 | 1.0 | 0.9 | 1.7 | 1.6 | 40414 | 40317 | 39971 | 40288 |
|  |  | KC1-P2-N_k25_Locus_3802_Transcript_72_1 | 21.6 | 31.3 | 39.0 | 35.1 | 4771 | 3240 | 2364 | 2697 |
|  |  | KC1-P2-N_k25_Locus_7273_Transcript_10_1 | 179.2 | 212.2 | 39.5 | 40.4 | 373 | 296 | 2324 | 2291 |
|  |  | KC1-P2-N_k31_Locus_8488_Transcript_3_1 | 4.3 | 6.6 | 2.4 | 3.1 | 19957 | 14227 | 33674 | 28888 |
|  |  | KC1-P2-N_k43_Locus_11606_Transcript_3_1 | 0.3 | 0.2 | 24.2 | 20.1 | 47352 | 48188 | 4210 | 5233 |
|  |  | KC1-P2-N_k43_Locus_18117_Transcript_1_1 | 3.5 | 3.6 | 3.3 | 3.4 | 22994 | 22451 | 27388 | 26964 |
|  |  | KC1-P2-N_k49_Locus_13283_Transcript_4_1 | 2.8 | 2.1 | 1.8 | 1.9 | 26745 | 31031 | 39076 | 37665 |
|  |  | KC1-P2-N_k61_Locus_9395_Transcript_6_1 | 3.5 | 4.2 | 11.0 | 10.1 | 23025 | 20110 | 9877 | 10713 |
|  |  | KC1-P2-N_k69_Locus_8612_Transcript_3_1 | 0.8 | 1.4 | 11.7 | 10.4 | 42791 | 35946 | 9278 | 10471 |
|  |  | KC1-P2_N1_CL4324Contig1_1 | 323.2 | 386.2 | 73.0 | 76.9 | 132 | 112 | 1028 | 972 |
|  |  | KC1-P2_N1_k49_Locus_7401_Transcript_1_1 | 7.7 | 11.3 | 6.9 | 5.8 | 12566 | 8886 | 15096 | 17469 |
|  |  | KC1-P2_N1_k55_Locus_365_Transcript_1_1 | 4.1 | 4.5 | 13.3 | 11.1 | 20568 | 18973 | 8180 | 9839 |
|  |  | KC1-P2_N2_k25_Locus_10816_Transcript_3_1 | 6.6 | 6.9 | 5.6 | 5.0 | 14468 | 13571 | 18061 | 19813 |
|  |  | KC1-P2_N3_k49_Locus_8953_Transcript_1_1 | 7.9 | 7.7 | 6.1 | 7.3 | 12370 | 12481 | 16956 | 14310 |
|  |  | KC1-P2_N4_k37_Locus_3169_Transcript_1_1 | 37.3 | 64.2 | 151.0 | 137.0 | 2610 | 1482 | 355 | 413 |

*ranks in the total 51,503 transcripts are shown.

**Supplementary Table 10.** List of the putative sugar uptake transporter in the *Braarudosphaera bigelowii* endosymbiont.

| Gene ID | Annotation | Blastp top hit | e-value |
| --- | --- | --- | --- |
| CPSB_00277 | sugar ABC transporter substrate-binding protein | *Candidatus* Atelocyanobacterium thalassa | 0 |
| CPSB_00506 | ABC transporter permease subunit | *Candidatus* Atelocyanobacterium thalassa | 1.00E-166 |
| CPSB_00347 | carbohydrate ABC transporter permease | *Candidatus* Atelocyanobacterium thalassa | 7.00E-170 |
| CPSB_00343 | ABC transporter ATP-binding protein | *Candidatus* Atelocyanobacterium thalassa | 0 |
| Annotation was performed using blastp to the NCBI-refprot database. | | |  |

**Supplementary Table 12.** Probabilities of trophic modes of *Braarudosphaera bigelowii* and *Haptolina brevifila*.

| Species | Accession | Phagocyte-generalist | Prototrophy | Photosynthesis |
| --- | --- | --- | --- | --- |
| *Haptolina brevifila* | MMETSP1094 | 0.98636 | 0.97980 | 0.95354 |
| *Brarrudosphaera bigelowii* | This study | 0.95430 | 0.99097 | 0.99309 |

**Supplementary Table 13.** List of putative secondary metabolites produced in *Crocosphaera watosonii* WH8501.

| Type | Number of the genomic regions | Most similar known cluster |
| --- | --- | --- |
| bacteriosin | 2 |  |
| NRPS-like | 1 | nematophin |
| NRPS-like | 1 | minutissamide A / minutissamide C / minutissamide D |
| NRPS | 2 | puwainaphycin A / puwainaphycin B / puwainaphycin C / puwainaphycin D |
| NRPS | 1 | anabaenopeptin NZ857/nostamide A |
| NRPS | 2 |  |
| NRPS | 1 | aeruginoside 126B / aeruginoside 126A |
| T1PKS, NRPS | 1 | aranazole A / aranazole B / aranazole C / aranazole D |
| NRPS, terpene | 1 |  |
| Annotation was performed using antiSMASH version 5.1.2 web server (Blin et al. 2019). | | |
